# An Integrated Systems Approach Unveils New Aspects of Microoxia-Mediated Regulation in *Bradyrhizobium diazoefficiens*

**DOI:** 10.3389/fmicb.2019.00924

**Published:** 2019-05-07

**Authors:** Noemí Fernández, Juan J. Cabrera, Adithi R. Varadarajan, Stefanie Lutz, Raphael Ledermann, Bernd Roschitzki, Leo Eberl, Eulogio J. Bedmar, Hans-Martin Fischer, Gabriella Pessi, Christian H. Ahrens, Socorro Mesa

**Affiliations:** ^1^Department of Soil Microbiology and Symbiotic Systems, Estación Experimental del Zaidín, Consejo Superior de Investigaciones Científicas, Granada, Spain; ^2^Agroscope, Research Group Molecular Diagnostics, Genomics and Bioinformatics and Swiss Institute of Bioinformatics, Wädenswil, Switzerland; ^3^Department of Health Sciences and Technology, Institute of Molecular Systems Biology, ETH Zurich, Zurich, Switzerland; ^4^Institute of Microbiology, ETH Zurich, Zurich, Switzerland; ^5^Functional Genomics Center Zurich, ETH & UZH Zurich, Zurich, Switzerland; ^6^Department of Plant and Microbial Biology, University of Zurich, Zurich, Switzerland

**Keywords:** comparative genomics, FNR/CRP proteins, genome sequencing, proteogenomics, post-transcriptional control, rhizobia, symbiosis, transcriptomics

## Abstract

The adaptation of rhizobia from the free-living state in soil to the endosymbiotic state comprises several physiological changes in order to cope with the extremely low oxygen availability (microoxia) within nodules. To uncover cellular functions required for bacterial adaptation to microoxia directly at the protein level, we applied a systems biology approach on the key rhizobial model and soybean endosymbiont *Bradyrhizobium diazoefficiens* USDA 110 (formerly *B. japonicum* USDA 110). As a first step, the complete genome of *B. diazoefficiens* 110*spc*4, the model strain used in most prior functional genomics studies, was sequenced revealing a deletion of a ~202 kb fragment harboring 223 genes and several additional differences, compared to strain USDA 110. Importantly, the deletion strain showed no significantly different phenotype during symbiosis with several host plants, reinforcing the value of previous OMICS studies. We next performed shotgun proteomics and detected 2,900 and 2,826 proteins in oxically and microoxically grown cells, respectively, largely expanding our knowledge about the inventory of rhizobial proteins expressed in microoxia. A set of 62 proteins was significantly induced under microoxic conditions, including the two nitrogenase subunits NifDK, the nitrogenase reductase NifH, and several subunits of the high-affinity terminal *cbb*_3_ oxidase (FixNOQP) required for bacterial respiration inside nodules. Integration with the previously defined microoxia-induced transcriptome uncovered a set of 639 genes or proteins uniquely expressed in microoxia. Finally, besides providing proteogenomic evidence for novelties, we also identified proteins with a regulation similar to that of FixK_2_: transcript levels of these protein-coding genes were significantly induced, while the corresponding protein abundance remained unchanged or was down-regulated. This suggested that, apart from *fixK*_2_, additional *B. diazoefficiens* genes might be under microoxia-specific post-transcriptional control. This hypothesis was indeed confirmed for several targets (HemA, HemB, and ClpA) by immunoblot analysis.

## Introduction

Rhizobia are primary contributors to biological nitrogen fixation, a process that is highly relevant both for agronomy and, by reducing the need of chemical fertilizers, for the environment. Optimizing the efficiency of nitrogen fixation is of great interest for sustainable agriculture and to potentially exploit symbiotic plant-microbe interactions for additional crops in the future. Consequently, there is a pressing need to further advance our understanding of the regulatory mechanisms that control rhizobia-leguminous plant interactions.

Rhizobia comprise a large group of both alpha- and beta-proteobacteria (reviewed in Sprent et al., 2017) which can establish symbiotic relationships with legumes. They encode the enzyme nitrogenase, which catalyzes the conversion of atmospheric nitrogen gas to ammonium inside plant root (and occasionally stem) nodules (reviewed in Dixon and Kahn, 2004; Terpolilli et al., 2012). During the establishment of a rhizobia-legume symbiosis, bacteria need to adapt their physiology from the free-living state in soil to the highly specialized environment of a plant cell, the so called bacteroid state. This multi-step process is tightly controlled at different stages of the symbiotic interaction and includes various signals released and sensed by both the bacteria and the host plant. One of these signals is the extremely low partial pressure of free oxygen (microoxia) within nodules. Microoxia represents a critical signal both for the expression and activity of nitrogenase and the *cbb*_3_-type high-affinity terminal oxidase required for bacterial respiration inside nodules (reviewed in Fischer, 1994, 1996; Dixon and Kahn, 2004; Terpolilli et al., 2012; Poole et al., 2018).

*Bradyrhizobium diazoefficiens* USDA 110 (formerly *B. japonicum* USDA 110; Delamuta et al., 2013) is one of the most important and best-studied rhizobial model species; it can form nodules on soybean (*Glycine max*) roots and a few other host plants like cowpea, mungbean, siratro, and others (reviewed in Sprent et al., 2017). Its molecular genetics, physiology, and ecology has been intensively investigated. The availability of several rhizobial genome sequences, including that of *B. diazoefficiens* USDA 110 (Kaneko et al., 2002; Davis-Richardson et al., 2016), has enabled functional genomics studies that have explored gene expression differences using either custom-made microarrays or RNA-Seq. Moreover, protein expression profiling studies using 2-D gels and later shotgun proteomics approaches provided further insights. The analysis of selected regulatory mutant strains, all grown under free-living microoxic conditions (Hauser et al., 2007; Lindemann et al., 2007; Pessi et al., 2007; Mesa et al., 2008), have greatly contributed to a better understanding of the regulatory mechanisms underlying the adaptation to the low oxygen tension encountered inside nodules. A complex regulatory network composed of two interlinked signaling cascades (FixLJ-FixK_2_ and RegSR-NifA) controls the expression of genes in response to microoxia, both in free-living conditions and in symbiosis (Sciotti et al., 2003; Pessi et al., 2007; reviewed in Fernández et al., 2016). For the transcription factor FixK_2_, which plays a key role in the microoxia-mediated regulation in *B. diazoefficiens* both in free-living conditions and in symbiosis, more than 300 regulated genes were identified including the *fixNOQP* operon, which encodes the *cbb*_3_-type high-affinity terminal oxidase (Mesa et al., 2008). The expression of *fixK*_2_ is induced by the superimposed two-component regulatory system FixLJ in response to microoxia, and is self-repressed by a yet unknown mechanism (reviewed in Fernández et al., 2016). FixK_2_ is a peculiar member of the cyclic AMP (cAMP) receptor protein (CRP) and the fumarate and nitrate reductase (FNR) activator protein family of bacterial transcription factors (Körner et al., 2003). It is active *in vitro* without additional effector molecules and is regulated post-translationally by the oxidation of its singular cysteine residue and by proteolysis (Mesa et al., 2005, 2009; Bonnet et al., 2013; reviewed in Fernández et al., 2016).

Due to the often modest correlation between gene expression and protein levels in bacteria, a comprehensive differential protein expression profiling of cells grown under microoxic conditions would complement the existing transcriptomics data and potentially uncover further aspects of the rhizobial adaptation to the nodule environment. However, while several proteomics studies exist on various stages of the rhizobial symbiosis (Winzer et al., 1999; Natera et al., 2000; Panter et al., 2000; Morris and Djordjevic, 2001; Djordjevic et al., 2003; Djordjevic, 2004; Sarma and Emerich, 2005; Larrainzar et al., 2007; Delmotte et al., 2010, 2014; Koch et al., 2010; Tatsukami et al., 2013; Clarke et al., 2015; Nambu et al., 2015; Marx et al., 2016; reviewed in Larrainzar and Wienkoop, 2017), data on the importance of microoxia in the adaptation to a nodule environment are scarce for rhizobial species. Two 2-D gel-based studies exist where protein expression patterns in oxic and low oxygen conditions were compared (Regensburger et al., 1986; Dainese-Hatt et al., 1999). The latter study had identified 24 of 38 differentially expressed proteins in cells grown under low oxygen (2% O_2_) or anaerobic conditions.

Notably, for *B. diazoefficiens*, most of the above functional genomics studies have in fact been performed with a spontaneous spectinomycin resistant derivative of *B. diazoefficiens* USDA 110 (*B. diazoefficiens* 110*spc*4; Regensburger and Hennecke, 1983). Since this strain was derived from the reference strain 36 years ago, it might well harbor genomic differences compared to the published USDA 110 NCBI reference genome sequence (NC_004463; Kaneko et al., 2002). In order to close this knowledge gap and to unravel new facets of the adaptation of *B. diazoefficiens* 110*spc*4 going from an oxic to a microoxic lifestyle, we applied an integrated systems approach. This included as first step a *de novo* genome assembly of the 110*spc*4 model strain, which revealed a deletion of 202 kb and several additional differences, that were not affecting symbiotic functions. We next performed a shotgun proteomics study using an adapted protocol of the gel-free, filter-aided sample preparation (FASP) methodology combined with liquid chromatography-tandem mass spectrometry (LC-MS/MS) and downstream bioinformatic data analysis, which included a detailed comparison of the genome of strain 110*spc*4 used in this study vs. that of the USDA 110 reference strain. Integration of protein expression data from oxic and microoxic conditions with those from previous transcriptomics experiments not only allowed us to identify proteins specifically induced under microoxic conditions, but also to discover new genes that are probably subject to post-transcriptional control like it was previously demonstrated for *fixK*_2_ (Mesa et al., 2009).

## Materials and Methods

### Bacterial Strains and Growth Conditions

*B. diazoefficiens* 110*spc*4 (wild type, a spontaneous spectinomycin-resistant derivative of *B. diazoefficiens* USDA 110), formerly *B. japonicum* USDA 110 (3I1b110; U.S. Department of Agriculture, Beltsville, MD, USA) (8,373 genes, 8,317 CDS), was routinely grown at 30°C in a peptone-salts-yeast extract (PSY) medium (Regensburger and Hennecke, 1983) as modified in Mesa et al. (2008). Oxic (21% O_2_) and microoxic cultures (0.5% O_2_, 99.5% N_2_) for shotgun proteomics, western blot analyses, and heme staining were grown to mid-exponential phase (optical density at 600 nm [OD_600_] of 0.45–0.6). For these experiments, oxic cultures were grown in 1-l Erlenmeyer flasks containing 50 ml of medium with vigorous shaking (170 rpm), while microoxic cultures were grown in 500 ml rubber-stoppered serum bottles containing 25 ml of medium with moderate shaking (60 rpm). In the latter, the gas phase was exchanged every 8–16 h. Spectinomycin was added to solid media at 200 μg/ml and liquid media at 100 μg/ml.

### Genome Sequencing, Assembly, and Annotation

*B. diazoefficiens* 110*spc*4 chromosomal DNA was isolated with a phenol/chloroform protocol as described previously (Hahn and Hennecke, 1984). PacBio SMRT sequencing was performed on an RSII machine using 1 SMRT cell. Size selection was performed using the BluePippin system which resulted in fragments with an average subread length of 13 kb. The PacBio reads were assembled using HGAP v.3, which was run on the SMRT Portal using the protocol “RS_HGAP_Assembly.3” (default parameters, except: minimum subread length of 1,000, estimated genome size of 9 Mb). The resulting contig was start-aligned with the RefSeq USDA 110 strain (NC_004463; Kaneko et al., 2002), and further polished with Quiver using the “RS_Resequencing.1” protocol (default parameters). To verify the circularity and completeness of the *de novo* assembly, the filtered PacBio subreads were mapped to the circular chromosome using graphmap (v.0.5.2) (Sović et al., 2016). The assembly was further improved using 2 × 300 bp paired end Illumina MiSeq reads and Freebayes (v.1.2.0; minimum alternate fraction: 0.5, minimum alternate count: 5) to correct small errors (e.g., homopolymer errors). Variants were manually inspected in the Integrated Genome Viewer (Thorvaldsdóttir et al., 2013) and subsequently corrected using bcftools (v.0.1.19) (Narasimhan et al., 2016). The genome was annotated with the prokaryotic genome annotation pipeline (Tatusova et al., 2016) used by the National Center of Biotechnology and Information (NCBI), which returned 8,407 genes, 8,348 CDS, and 248 pseudogenes. A functional classification of the genes in Clusters of Orthologous Groups (COG) categories was performed using eggNOG and the database “bactNOG,” “proNOG,” and “aproNOG” (Huerta-Cepas et al., 2016). Best hits with an e-value below 0.001 were used to assign the COG category. In addition, all protein-coding genes were also annotated using Interproscan v5.30-69.0 (Jones et al., 2014) to add information on protein domains, families, and patterns, PSORTb (Yu et al., 2010) to identify a predicted subcellular localization, and LipoP (Rahman et al., 2008) to identify lipoproteins. Further, proteins were classified as transmembrane (TM), secreted or membrane anchored using a combination of TM spanning helices and signal peptides predicted by TMHMM v2.0, SignalP v4.1, and Phobius v1.01 (predictions were extracted from Interproscan results), basically as described before (Delmotte et al., 2010) except that we considered a cutoff of 2 TM domains. We only considered cases where the predictions for TM helices and signal peptides of the two tools overlapped by at least 10 amino acids.

### Comparative Genomics

**Figure 1:**
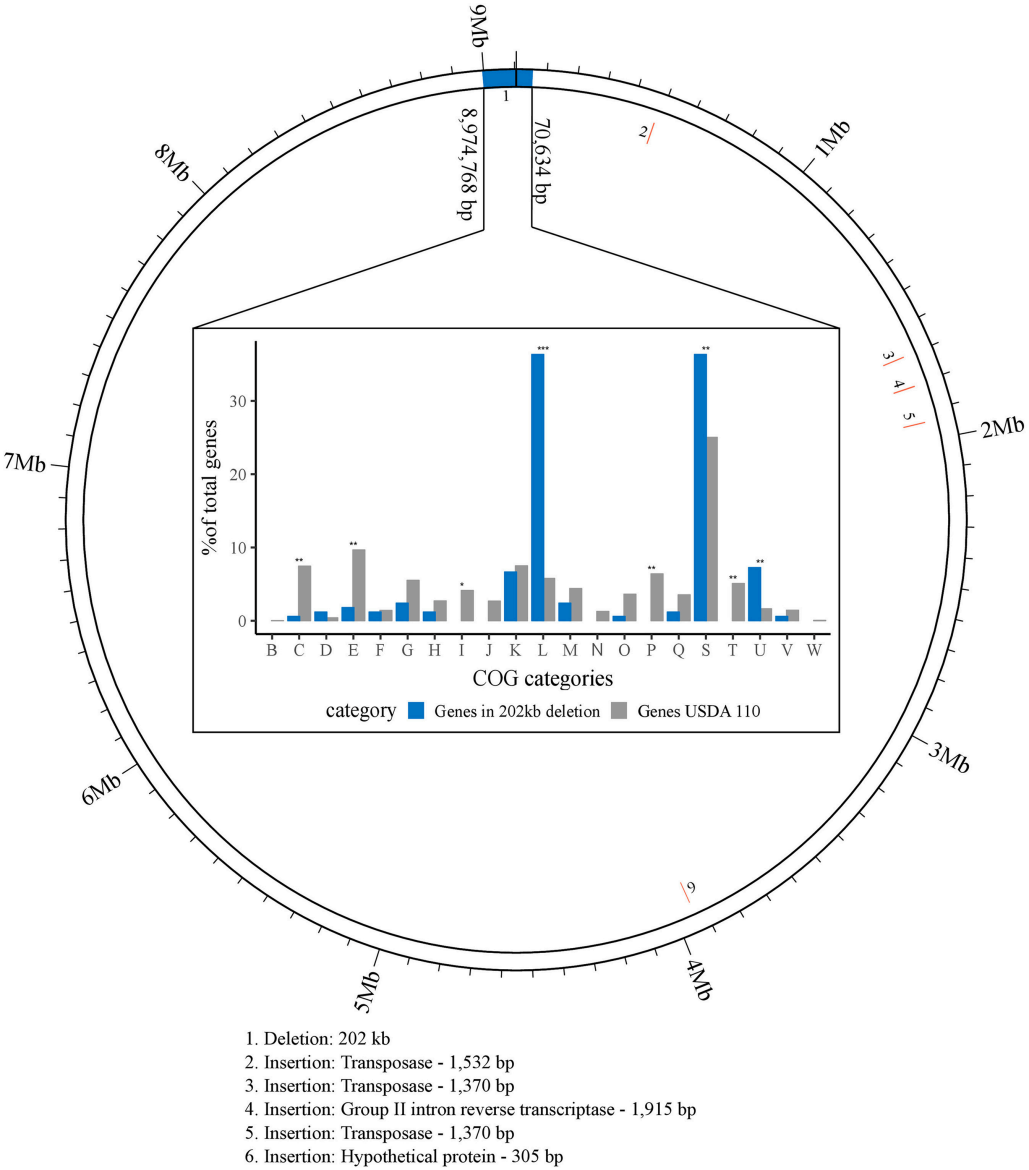
Visualization of larger genomic changes in *B. diazoefficiens* strain 110*spc*4 (inner ring) compared to the USDA 110 NCBI reference genome (outer ring). Five insertions (2 through 6; red) and a large deletion region (1; blue) are shown (see Table 1). The bar chart shows the results of a Fisher's exact test for enrichment of COG categories annotated in 142 of 223 genes located in the 202 kb region of USDA 110 that is absent in strain 110*spc*4. Significant *p*-values are highlighted above each category (^*^p-value < 0.01; ^**^p-value < 0.001; ^***^p-value < 0.00001). The percentages of genes (count of genes / total no. of genes) for each COG category (X-axis) from USDA 110 (gray) or from the 202 kb deletion region (blue) are shown on the Y-axis.

The genome sequence of *B. diazoefficiens* 110*spc*4 was aligned to that of the USDA 110 RefSeq strain using the progressive Mauve algorithm (v.2.4.0) (Darling et al., 2010) in order to inspect for larger-scale structural variations. Furthermore, it was mapped to the RefSeq genome sequence using Minimap2 (v.2.10; preset parameters: asm5) (Li, 2018) and smaller variants were detected using Freebayes (v.1.2.0; minimum alternate fraction: 0.1, minimum alternate count: 1). SnpEff (v.4.3) (Cingolani et al., 2012) was used to annotate and predict potential effects of the detected variants in strain USDA 110. For this, a database of the RefSeq genome was built using the annotated GenBank file, which was subsequently used to interpret variants. Related data summarizing major genomic differences are shown in Figure 1; Table 1 and Supplementary Tables S1, S2. Finally, we also compared the proteins encoded by the two genomes using blastp (Johnson et al., 2008). Only hits below an e-value of 1e-5 were considered. For proteins with multiple hits, we considered the hit with the highest amino acid identity and query coverage, respectively (Supplementary Table 3, Data Sheet A). All references cited in the Supplementary Material are listed in Supplementary Data Sheet 1.

**Table 1:** Overview of larger genomic differences between strain *B. diazoefficiens* 110*spc*4 compared to the NCBI *B. diazoefficiens* USDA 110 reference genome (see also Figure 1).

**Change in 110*spc*4**	**Start pos. in USDA 110 (RefSeq)**	**Start pos. in 110*spc*4**	**Length (bp)**	**Gene(s) affected or contained in inserted region**	**Gene, product interrupted (CDS in reference)**
DEL	1	1	70,634	78 CDS	Together with the deletion below (next row), 223 CDS are affected. See Supplementary Table S1
DEL	8,974,768	8,910,608	131,060	145 CDS	Together with the deletion above (previous row), 223 CDS are affected. See Supplementary Table S1
INS	483,970	413,137	1,532	Transposase (Bdiaspc4_01920)	No CDS in USDA 110 reference
INS	1,699,880	1,630,578	1,370	Transposase (Bdiaspc4_07865)	No CDS in USDA 110 reference
INS	1,806,987	1,739,052	1,915	Group II intron reverse transcriptase (Bdiaspc4_08330)	No CDS in USDA 110 reference
INS	1,936,754	1,870,731	1,370	Transposase (Bdiaspc4_09015)	Bll1778^a^, unknown protein (1,936,357–1,937,313)
INS	3,936,542	3,872,887	305	Hypothetical protein (Bdiaspc4_18405)	Bll3563^b^, unknown protein (3,930,196–3,940,521)

a Bll1778 is a hypothetical protein of 318 amino acids; it has no PFAM-A protein domains.

b Bll3563 is a large protein (3441 aa) with many VCBS domains (suggested role in adhesion).

*DEL, deletion; INS, insertion*.

### Sample Preparation for Shotgun Proteomics

Three replicates of 100 ml of culture grown to mid-exponential phase under oxic and microoxic conditions were collected by centrifugation (11,139 × *g* for 5 min at 4°C), and pellets were kept at −80°C. Each pellet was resuspended in 1 ml of 125 mM Tris-HCl pH 8.2 buffer supplemented with a protease inhibitor cocktail (cOmplete, Roche Diagnostics) according to the manufacturer's recommendations, before the cell suspension was sonicated (1 min, cycle 5, 50% amplitude; Bandelin UW2070). The above procedure was followed by disruption through an ice-cold French pressure cell (SLM Aminco) at about 120 MPa, and another round of sonication (same settings). One-hundred μl lysate per sample were mixed with 4% sodium dodecyl sulfate (SDS) and 0.1 M dithiothreitol (DTT) and boiled at 95°C for 6 min with gentle shaking (at 700 rpm) in a thermomixer comfort (Vaudaux-Eppendorf AG). Samples were then processed with an ultrasonic cup horn (UTR200, Heilscher Ultrasonics GmbH) for 15 min at an amplitude of 65% for a 0.5 cycle and centrifuged for 10 min at 16,000 × *g*. Protein concentration was determined using the Qubit^TM^ Protein Assay Kit (Invitrogen Life Technologies). Twenty microgram protein of each sample were used for on-filter digestion using a modified version of the FASP protocol (Wiśniewski et al., 2009a,b). Proteins were diluted in 200 μl of 100 mM Tris-HCl pH 8.2 buffer containing 8 M urea (UA buffer), loaded on Ultracel 30,000 MWCO centrifugal units (Amicon Ultra, Merck), and centrifuged at 14,000 × *g* for 20 min. The loaded filter was washed once with 200 μl UA buffer at 14,000 × *g* for 15 min. Alkylation of reduced proteins was carried out by adding 100 μl 0.05 M iodoacetamide in UA buffer shaking for 1 min at 600 rpm in a thermomixer, followed by 5 min incubation at room temperature and centrifugation at 14,000 × *g* for 20 min. Then, samples were washed with 100 μl UA buffer three times (centrifugation at 14,000 × *g* for 15 min), followed by two washing steps with 100 μl 0.5 M NaCl in water. On-filter protein digestion was carried out by adding 120 μl trypsin containing triethylammonium bicarbonate buffer (pH 8.5) (Promega) at a ratio of 1:50 (w/w) onto the filter, mixing for 1 min in a thermomixer at 600 rpm, and overnight incubation in a wet chamber at room temperature. The filter units were then centrifuged at 14,000 × *g* for 20 min and the peptide containing solution was acidified to a final concentration of 0.1% trifluoroacetic acid and 3% acetonitrile. Peptides were desalted using Finisterre C18 SPE cartridges (Teknokroma) following the manufacturer's protocol. Eluted peptides were dried in a centrifugal vacuum concentrator (Thermo Servant SPD 121P) and dissolved in 50 μl of 3% acetonitrile and 0.1% formic acid for mass spectrometry (MS) analysis.

### Liquid Chromatography-Tandem Mass Spectrometry (LC-MS/MS) Analysis

Dissolved peptides were separated on a self-made reverse-phase column (75 μm × 150 mm) packed with C18 material (ReproSil-Pur, C18, 120 Å, AQ, 1.9 μm, Dr. Maisch GmbH). The column was equilibrated with 100% solvent A (0.1% formic acid in water). Peptides were eluted using the following gradient of solvent B (0.1% FA in acetonitrile): 0–50 min, 0–25% B; 50–60 min, 25–32% B; 60–70 min, 32–97% B at a flow rate of 0.3 μl/min. High accuracy mass spectra were acquired with an Orbitrap Fusion (Thermo Scientific) that was operated in data-dependent acquisition mode. All precursor signals were recorded in the Orbitrap using quadrupole transmission in the mass range of 300–1,500 m/z. Spectra were recorded with a resolution of 120,000 at 200 m/z, a target value of 4E5 and the maximum cycle time was set to 3 s. Data-dependent MS/MS were recorded in the linear ion trap using quadrupole isolation with a window of 1.6 Da and HCD fragmentation with 30% fragmentation energy. The ion trap was operated in rapid scan mode with a target value of 2E3 and a maximum injection time of 300 ms. Precursor signals were selected for fragmentation with a charge state from +2 to +7 and a signal intensity of at least 5E3. A dynamic exclusion list was used for 30 s and maximum parallelizing ion injections was activated.

### Protein Identification, Differential Protein Expression Analyses, and Integration With Transcriptomics Data

Data from three LC MS/MS runs per condition were processed with an in-house data analysis pipeline (Omasits et al., 2013). Raw data were converted with msconvert (ProteoWizard release 3.09.9098) before extracting fragment-ion spectra. A search against a *B. diazoefficiens* USDA 110 (8,317 CDS) and a 110*spc*4 (8,348 CDS) protein search database (both also containing 256 common contaminants) was carried out with MS-GF+ (v2017.01.13) (Kim and Pevzner, 2014) using a precursor mass accuracy of 10 ppm (the fragment ion mass tolerance is determined by the algorithm). Cysteine carbamidomethylation as fixed, oxidation of methionine and deamidation of asparagine and glutamine as variable modifications were set. Using the target-decoy approach of MS-GF+, the false discovery rate (FDR) at the peptide-spectrum-matching (PSM) level was estimated and then set below 0.35%. For protein inference, we only considered unambiguous peptides (class 1a, class 3a) as identified by a PeptideClassifier analysis (Qeli and Ahrens, 2010). Requiring at least two peptides or three PSMs per condition per protein, i.e., the same thresholds as used previously (Delmotte et al., 2010; Koch et al., 2014), 3,188 proteins were identified overall for strain 110*spc*4 with a protein level FDR below 1%. To detect differentially expressed proteins, we used DESeq2 (Love et al., 2014) which returns a list of proteins ranked according to their statistical significance. A multiple testing corrected *p*-value threshold of ≤ 0.2 was applied as selection criterion for the identification of the top significantly expressed proteins. Since previous transcriptomics data were filtered based on a fold change (FC) expression difference level (log_2_ FC ≥1 or ≤ −1), we applied the same, less stringent filter for the further overlap analysis of genes and proteins upregulated in microoxia (log_2_ FC ≥ 1).

### Western Blot Analyses

Steady-state levels of *B. diazoefficiens* NapA, and NosZ (microoxia-induced proteins) and ClpA, FixK_2_, HemA, and HemB (the corresponding protein-encoding genes are subject to post-transcriptional control) were monitored in cells grown oxically and microoxically by immunoblot analysis. Proteins with constant accumulated levels in both oxic and microoxic conditions, i.e., ClpP, CoxA, CoxB, and ScoI were used as controls in the experiments. At least three biological replicates of two hundred milliliters of mid-exponential grown oxic and microoxic cultures were collected by centrifugation (9,000 × *g*, 7 min, 4°C), resuspended in 2 ml of lysis buffer (40 mM Tris-HCl pH 7.0, 150 mM KCl, 0.2 mM 4-[2-Aminoethyl] benzenesulfonyl fluoride hydrochloride [AEBSF]) and disrupted by four passages through an ice-cold French pressure cell (SLM Aminco) at about 120 MPa. Cell suspensions were centrifuged (21,000 × *g*, 30 min, 4°C) to obtain total cell-free extracts. For fractionation, cell-free extracts were then ultracentrifuged at 140,000 × *g* for 45 min at 4°C. Membrane pellets were resuspended in 100 μl of lysis buffer. Ten to forty micrograms of protein (crude extract, soluble fraction, or membranes) were then mixed with one sixth volume of SDS loading dye (280 mM Tris-HCl, pH 6.8, 20% glycerol, 8% SDS, 480 mM DTT, 0.02% bromophenol blue). For ClpA, CoxB, and HemB detection, protein samples were mixed with an equal volume of SDS loading dye containing mercaptoethanol (125 mM Tris-HCl, pH 6.8, 20% glycerol, 4% SDS, 10% mercaptoethanol, 0.02% bromophenol blue). Proteins were subsequently resolved in 14% sodium dodecyl sulfate polyacrylamide gel electrophoresis (SDS-PAGE), and analyzed by western blotting basically as previously described by Torres et al. (2017) (except for ClpA, CoxB, and HemB detection, 1% casein instead of 5% non-fat dry milk was added in the blocking buffer in our experiments). Samples were boiled prior to electrophoresis except for CoxA detection, where samples were equilibrated for 15 min in SDS loading dye. Polyclonal antibodies against FixK_2_, HemA, and HemB of *B. diazoefficiens* (Chauhan and O'Brian, 1995; Jung et al., 2004; Mesa et al., 2009), ClpA of *Caulobacter crescentus* (Grünenfelder et al., 2004), NosZ of *Paracoccus denitrificans* (Felgate et al., 2012), and NapA of *P. pantotrophus* M6 (Gates et al., 2003) were available from previous work. A polyclonal antibody against *C. crescentus* ClpP (Jenal and Fuchs, 1998), peptide antibodies raised against *B. diazoefficiens* CoxA (Loferer et al., 1993) and *B. diazoefficiens* CoxB (Bühler et al., 2010), and a polyclonal antibody against the soluble part of *B. diazoefficiens* ScoI (Bühler et al., 2010) were used to monitor protein levels in extracts of oxic and microoxic cultures. Except for *C. crescentus* anti-ClpP (1:5,000 dilution), *B. diazoefficiens* anti-CoxB (1:10,000 dilution), and *B. diazoefficiens* anti-HemB (1:2,000 dilution) primary antibodies were diluted at 1:1,000 in blocking buffer. Horseradish peroxidase (HRP)-conjugated goat anti-rabbit IgG (for ClpA, ClpP, CoxA, CoxB, FixK_2_, HemB, NapA, and ScoI; Bio-Rad), HRP-conjugated goat anti-mouse IgG (for HemA; Bio-Rad), and HRP-conjugated donkey anti-sheep (for NosZ; Sigma-Aldrich) were used as secondary antibodies at a 1:3,000 dilution. Chemiluminescent signals were detected using the ECL Select western-blotting detection reagent (GE Healthcare) in a Chemidoc XRS instrument (Universal Hood II, Bio-Rad). The Quantity One and Image Lab softwares (Bio-Rad) were used for image analyses.

### Cytochrome *c* Detection by Heme Staining

Membrane-bound heme-*c* protein detection was performed as described elsewhere (Delgado et al., 2003; Mesa et al., 2008; Bueno et al., 2010). Two hundred milliliters of *B. diazoefficiens* cells grown oxically and microoxically were harvested at mid-exponential phase, washed with 50 mM Tris-HCl buffer (pH 7.5) (fractionation buffer) and resuspended in 2 ml of the same buffer containing 1 mM AEBSF, and 10 μg DNase I/ml. Cell fractionation was performed as described above. Membrane pellets were resuspended in 100 μl of fractionation buffer. Membrane fractions were mixed with one sixth volume of SDS loading dye with reduced DTT concentration (20 mM instead of 480 mM), incubated at room temperature for 15 min and loaded without boiling onto 12% SDS-PAGE. Proteins were then transferred to a nitrocellulose filter and stained for heme-dependent peroxidase activity by chemiluminescence (Vargas et al., 1993), which was detected and analyzed as described above for the western blot assays. Experiments were carried out with at least two biological replicates.

### Determination of Protein Concentration

Protein concentration of samples for western blot and heme staining was estimated spectrophotometrically using the Bio-Rad assay (Bio-Rad Laboratories) with bovine serum albumin (BSA) as standard.

### Plant Infection Test and Determination of Nitrogenase Activity

Seeds of soybean (*G. max* (L.) Merr.) cv. Williams 82 (kindly provided by D.-N. Rodríguez Navarro, CIFA, Las Torres-Tomejil, Seville, Spain), and cv. Black Jet, cowpea (*Vigna unguiculata* (L.) Walp.) cv. Iron and Clay, mung bean (*Vigna radiata* (L.) R. Wilczek) (all purchased from Johnny's Selected Seeds, Albion, ME, USA), and siratro (*Macroptilium atropurpureum* (DC.) Urb. provided by W. D. Broughton, University of Geneva, Switzerland) were surface-sterilized by immersion in 100% ethanol for 5 min and subsequently in 35% H_2_O_2_ for 15 min. After intense washing with sterile water, seeds were germinated for 48 h at 28°C. Sterilization and germination of *Aeschynomene afraspera* seeds was done as described (Renier et al., 2011). Plant growth conditions were described previously (Göttfert et al., 1990). *A. afraspera* plants were cultivated as all other plants, except that the substrate was kept water-logged.

Nitrogenase activity was determined by gas chromatographic detection of ethylene (C_2_H_4_) resulting from acetylene (C_2_H_2_) reduction by nitrogenase. To do so, whole roots of nodulated plants were incubated in 50-ml sealed vials and 1 ml acetylene (2% [v/v]) was added to the gas atmosphere. After incubation for 30 min at room temperature, 25 μl of the gas phase were analyzed on a GC6850 gas chromatograph (Agilent Technologies) equipped with a 30 m × 0.53 mm HP-PLOT/Q column. Enzyme activity (units; calculated by dividing the C_2_H_4_ peak area by the sum of the C_2_H_4_ + C_2_H_2_ peak area) was normalized to the total nodule dry weight of individual plants and 1 min incubation time.

## Results

### Sequencing and *de novo* Genome Assembly of *B. diazoefficiens* Strain 110*spc*4

Many gene and protein expression profiling studies of *B. diazoefficiens* cells grown under free-living conditions or in symbiosis (Lardi and Pessi, 2018 and Discussion) have been performed with *B. diazoefficiens* 110*spc*4 (Regensburger and Hennecke, 1983). Given the split from the USDA 110 reference strain 36 years ago, strain 110*spc*4 might well harbor genomic differences compared to the 9,105,828 bp genome of the reference strain (NC_004463; Kaneko et al., 2002). Aiming to provide an optimal basis for subsequent functional genomics and systems-wide analyses for strain 110*spc*4, we sequenced and *de novo* assembled its complete genome using a combination of long PacBio reads (average read length 13 kb) and short Illumina MiSeq reads (paired-end, 300 bp). The latter were used to correct any potentially remaining homopolymer errors in the PacBio assembly. The finished, high-quality genome sequence of *B. diazoefficiens* 110*spc*4 consisted of a single, circular chromosome of 8,910,608 bp. A genome comparison uncovered several differences compared to the USDA 110 reference genome (Table 1), most notably a 202 kb deletion of a genomic region corresponding to nucleotides 1–70,634 and 8,974,768–9,105,828 of the USDA 110 reference genome sequence (Figure 1). This 202 kb deletion, which harbors 223 CDS (Supplementary Table S1), was confirmed by a PCR analysis (Supplementary Figure S1). The genome alignment further revealed insertion of genes for three transposases, one group II intron transcriptase and one hypothetical protein in the 110*spc*4 genome (Figure 1). These insertions did either not affect a CDS or occurred in genes encoding proteins of unknown function (Table 1). Among additional changes based on smaller differences (insertion or deletion of one nucleotide, non-synonymous substitutions), 26 were predicted to lead to a frameshift in a CDS (Supplementary Table S2, see below).

**Figure 2:**
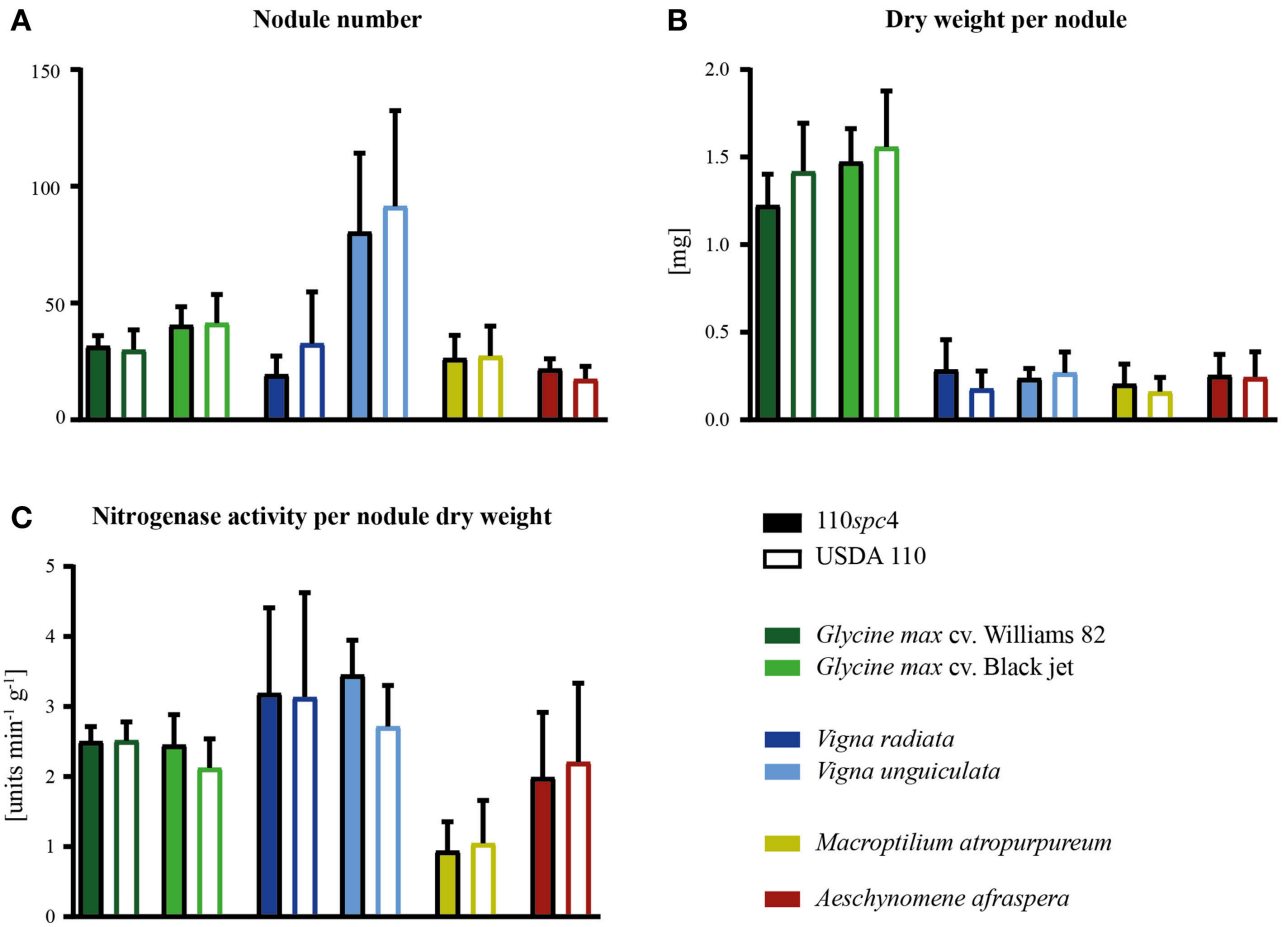
Symbiotic properties of *B. diazoefficiens* strains 110*spc*4 and USDA 110 on different legume host plants. No significant difference was found between strains 110*spc*4 and USDA 110 with respect to nodule number **(A)**, dry weight per nodule **(B)**, or nitrogenase activity normalized to total nodule dry weight **(C)** on all six tested host plants, following a one way ANOVA with Holm-Šídáks correction (0.05 α). The two soybean (*Glycine max*) cultivars Williams 82 (*n* = 9 and 10 for 110*spc*4 and USDA 110, respectively) and Black jet (*n* = 10 and 10), mung bean (*Vigna radiata*; *n* = 9 and 7), cowpea (*Vigna unguiculata, n* = 8 and 8), and the crack-entry host *Aeschynomene afraspera* (*n* = 10 and 9) were analyzed 21 days post inoculation (dpi) whereas siratro plants (*Macroptilium atropurpureum, n* = 8 and 10) were analyzed 28 dpi. Error bars represent standard deviation of the mean.

Importantly, a test carried out with six major host plants of *B. diazoefficiens*, namely two soybean (*G. max*) cultivars (Williams 82, Black jet), mungbean (*Vigna radiata*), cowpea (*Vigna unguiculata*), siratro (*Macroptilium atropurpureum*), and the crack-entry host *Aeschynomene afraspera*, revealed that none of the four analyzed parameters relevant for symbiosis (nodule number, dry weight per nodule, nitrogenase activity, leaf color) were significantly affected by the genomic deletion in strain 110*spc*4 compared to the USDA 110 reference strain (Figure 2; Supplementary Figure S2). A Fisher's exact test of COG categories indicated a significant under-representation of several important functional categories among the 223 deleted genes compared to all genes. These included categories C (energy production and conversion), E (amino acid transport and metabolism), I (lipid transport and metabolism), P (inorganic ion transport and metabolism), and T (signal transduction mechanisms) (Figure 1). Conversely, genes in category U (intracellular trafficking, secretion, and vesicular transport), S (function unknown) and most prominently in category L (replication, recombination and repair), which includes many transposases, were significantly enriched. These genes apparently do not play an important role in symbiosis.

### Determination of the Proteome Expressed Under Oxic and Microoxic Conditions

**Figure 3:**
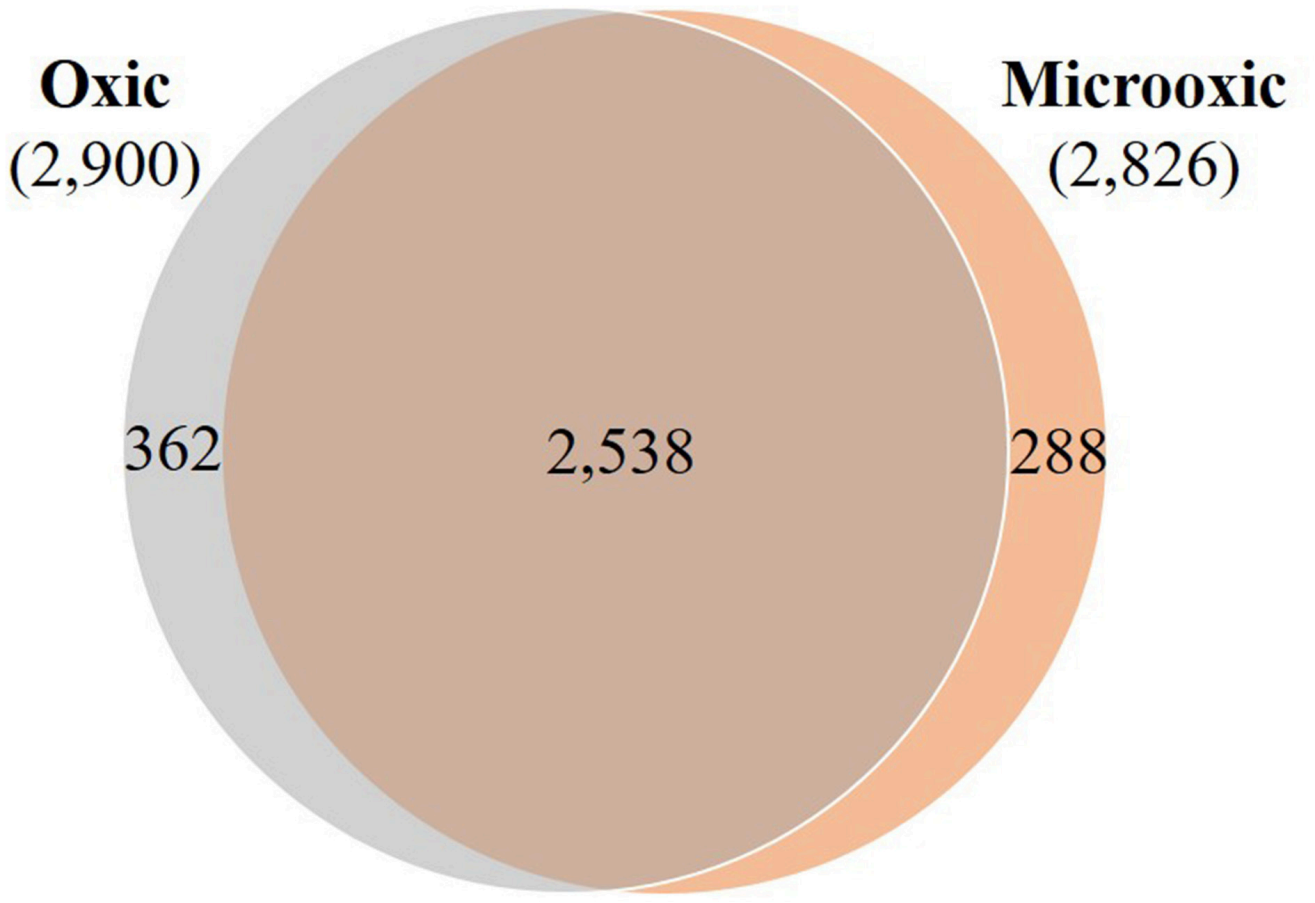
Overlap of proteins whose expression was identified in oxic and microoxic conditions. Venn diagram representing the proteins identified in oxic (2,900) and microoxic (2,826) conditions, their overlap (2,538), as well as microoxia-specific (288) and oxia-specific (362) proteins.

With the *de novo* assembled and annotated genome of our model strain *B. diazoefficiens* 110*spc*4 at hand, we next aimed to identify the set of proteins differentially expressed in microoxia, which has been recognized as a key signal for the induction of symbiosis-related genes during different steps of bacteria-host plant interaction (Terpolilli et al., 2012; Poole et al., 2018). Therefore, we performed a shotgun proteomics approach based on the gel-free FASP technology (Wiśniewski et al., 2009a,b) of cells grown under microoxic and oxic conditions (three replicates each; Supplementary Figure S3). Using a stringent FDR at the PSM level (0.35%) and requiring two distinct peptides or three PSMs of unambiguous peptides (Qeli and Ahrens, 2010) per condition, i.e., the same thresholds as used in previous studies (Delmotte et al., 2010; Koch et al., 2014), we were able to detect 3,188 expressed proteins (including two cases of 3a protein groups; i.e., identical protein sequences encoded by different genetic loci; Supplementary Table S3, Data Sheet B). The overall estimated FDR at the protein level was below 1%. 2,900 and 2,826 proteins were identified in oxic and microoxic conditions, respectively (Figure 3; Supplementary Table S3, Data Sheets C,D), corresponding to roughly 34% each of the 8,348 proteins encoded by the *B. diazoefficiens* 110*spc*4 genome. A search against the USDA 110 reference genome database returned virtually identical results. As expected, none of the protein products of the 223 genes located in the deletion region of 110*spc*4 were detected by unambiguous peptide evidence. Among the 26 genes affected by a frameshift (single nucleotide insertion or deletion), 9 protein products were expressed (Supplementary Table S2). It has to be noted that the NCBI genome annotation pipeline annotated more CDS (8,348) in the smaller genome of strain 110*spc*4 compared to the USDA 110 reference strain (8,317 CDSs). To enable the *Bradyrhizobium* research community to carry out similar comparative analyses, we have included Supplementary Table S3, Data Sheet A, which lists for each of the 8,348 annotated *B. diazoefficiens* 110*spc*4 proteins the respective best Blast hit in the USDA 110 genome along with the locus tag information, additional functional annotations, and other metadata.

Of the 2,826 proteins expressed under microoxic conditions, 2,538 proteins were also identified in cells cultured under oxic conditions (Figure 3). However, a group of 288 proteins was exclusively expressed under microoxic conditions. Previous transcriptomics experiments performed with a customized tiling-type GeneChip array designed based on the USDA 110 reference genome had revealed that 5,568 and 5,439 genes were transcribed in microoxically and oxically grown cells, respectively (Pessi et al., 2007), and that 506 genes were specifically transcribed under microoxic conditions. While minor genomic annotation differences might affect a few of these genes, we relied on Supplementary Table S3 (Data Sheet A) and focused on matching all transcribed USDA 110 protein-coding genes to their respective counterpart in strain 110*spc*4 (via a best Blast hit analysis at the protein level). Following this approach, 72 proteins of 506 microoxia-specific genes were present among the 288 microoxia-specific proteins identified in this study (Supplementary Figure S4). Another 75 of the microoxia-specific genes were detected at the protein level under oxic conditions. Considering an additional eight annotation differences between the two strains (see Supplementary Figure S4), the remaining 639 genes/proteins represent the set of microoxia-specific expressed genes and proteins (Supplementary Table S3, Data Sheet E).

### Comparison of Differential Protein and Gene Expression Under Microoxic Conditions

**Figure 4:**
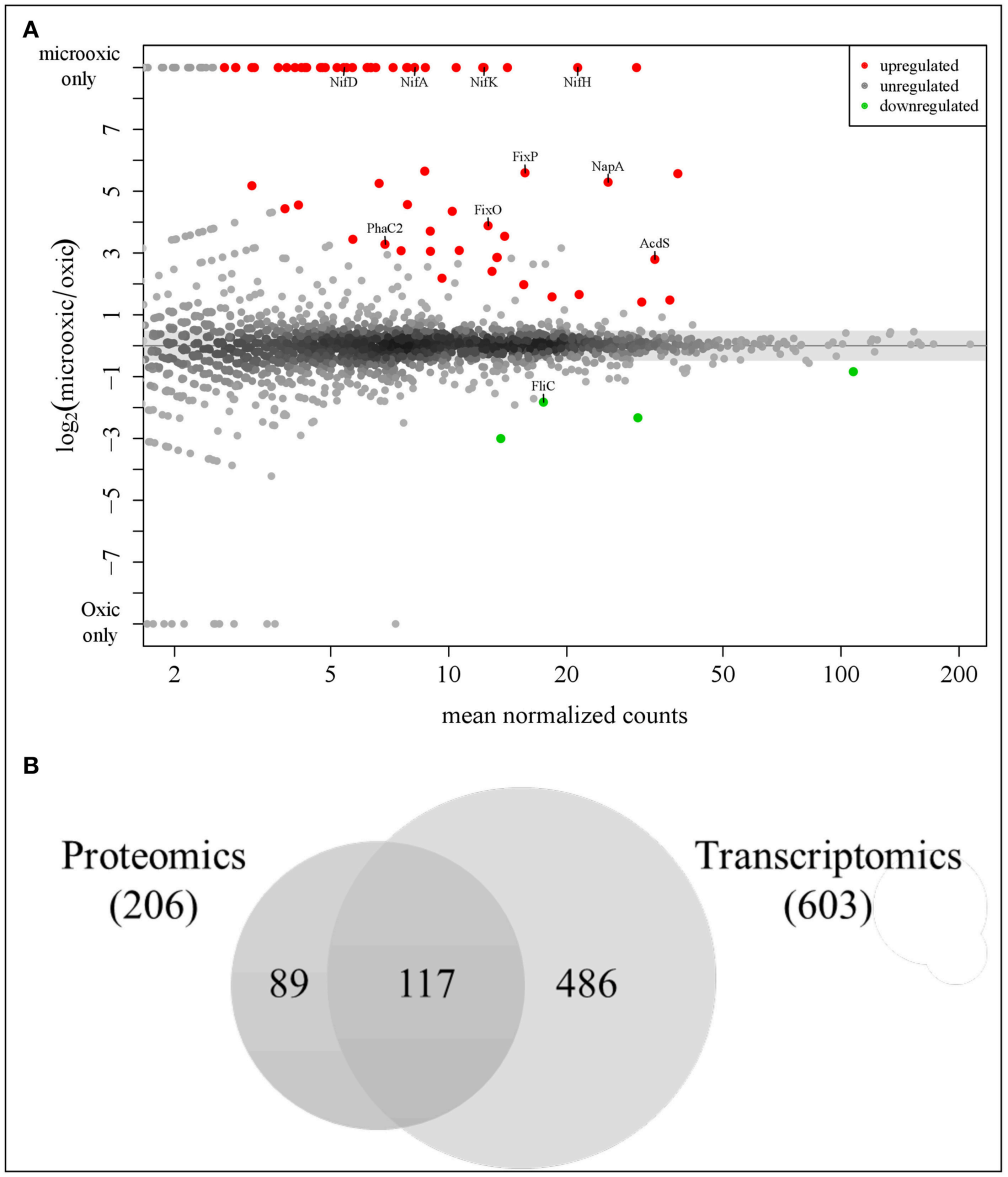
Analysis of differential protein and gene expression in microoxia. **(A)** MA plot visualizing the differential protein expression data. Sixty-two proteins most significantly up-regulated in microoxia are shown in red, those most significantly down-regulated (four proteins) in green. The names of selected proteins are indicated. **(B)** Overlap of proteins upregulated in microoxic conditions with previous transcriptomics data (Pessi et al., 2007). Using a less stringent threshold (log_2_ FC ≥ 1) identical to that used in a prior transcriptome analysis, 206 proteins fulfilled the criterion, including the top 62 up-regulated proteins (*p*-value ≤ 0.2) shown in **(A)**. The overlap with 603 protein-coding genes among the 620 genes previously found to be induced at the transcript level (Pessi et al., 2007) is shown here: 117 genes/proteins are upregulated both at transcript and protein level (see Table 2).

To identify proteins specifically induced in cells grown under microoxic conditions compared to oxic conditions, we used DESeq2 (Love et al., 2014). By applying a multiple testing corrected *p*-value threshold of ≤ 0.2, the comparison resulted in a list of top 66 significantly differentially expressed proteins (Figure 4A). These included 62 proteins that were induced under microoxic conditions (“core microoxic proteome”) and four proteins whose expression was significantly downregulated under microoxic conditions, respectively (Table 2). Among the top upregulated proteins, we found many of the prominent targets characteristic for the microoxic lifestyle, e.g., the two subunits of the nitrogenase (NifD and NifK) and nitrogenase reductase (NifH), two subunits of the *cbb*_3_ high-affinity terminal oxidase (FixO, FixP), the NifA transcription regulator, one of the five polyhydroxybutyrate polymerase paralogs (PhaC2), or the 1-aminocyclopropane-1-carboxylate (ACC) deaminase protein (AcdS) involved in degradation of the ethylene intermediate ACC (Ma et al., 2002; Murset et al., 2012). The overlap between the 62 proteins and the previously defined microoxia-induced transcriptome of 620 genes (17 of which are non-protein coding) (Pessi et al., 2007) led to the definition of 46 genes/proteins induced under microoxic conditions (Table 2). Further, by comparing the 62 upregulated proteins with the group of genes activated by two of the main regulatory proteins (NifA, FixK_2_) that respond to microoxic conditions in *B. diazoefficiens*, we found that 18 protein-encoding genes overlap with the combined NifA + RpoN_1/2_ regulon which consists of 65 genes (Hauser et al., 2007) (Table 2). RpoN is an alternative sigma factor that functions in concert with the regulatory protein NifA for the activation of the expression of nitrogen fixation-related genes (*nif* , *fix*) among others (reviewed in Dixon and Kahn, 2004). Interestingly, 13 protein-encoding genes are also part of the described putative direct targets for FixK_2_ (Mesa et al., 2008) (Table 2). Notably, ~89% of the genes coding for the 62 microoxia-induced proteins (i.e., 55 genes), were also shown to be upregulated in soybean nodules (Pessi et al., 2007) (Table 2).

**Table 2:** List of the top 66 significantly differentially expressed proteins in *B. diazoefficiens* cells grown under microoxic conditions (0.5% O_2_) in comparison to oxic conditions (62 proteins upregulated that constitute the “core microoxic proteome,” and four proteins downregulated; multiple testing corrected *p*-value ≤ 0.2).

**Locus_tag^a^**	**Product^b^**	**Gene name^c^**	**Query^d^**	**log_**2**_ FC (microoxic vs. oxic)^e^**	**FC (microoxic vs. oxic)^f^**	**FC (soybean bacteroids vs. oxic)^g^**	**NifA+RpoN_**1**_ targets^h^**	**FixK_**2**_ targets^i^**
**Sixty-two proteins showing increased expression in microoxic conditions in comparison to oxic conditions (*****p*****-value** **≤** **0.2)**								
Bdiaspc4_00855	Aminocyclopropane-1-carboxylate deaminase/D-cysteine desulfhydrase family protein	*acdS*	blr0241	2.8	4.8	20.1	–	–
Bdiaspc4_05275	Benzoyl-CoA-dihydrodiol lyase	–	blr1080	3.1	–	25.4	–	–
Bdiaspc4_06495	Propionyl-CoA synthetase	*acs*	blr1309	1.6	3.1	–	–	–
Bdiaspc4_06505	OmpW family protein	–	blr1311	6.0	44.9	23.3	–	+
Bdiaspc4_07020	ATP-dependent chaperone ClpB	*clpB*	blr1404	1.5	2.7	3.0	–	–
Bdiaspc4_08825	Nitrogenase molybdenum-iron protein alpha chain	*nifD*	blr1743	6.0	13.8	107.3	+	–
Bdiaspc4_08830	Nitrogenase molybdenum-iron protein subunit beta	*nifK*	blr1744	7.1	12.4	106.1	+	–
Bdiaspc4_08835	Nitrogenase iron-molybdenum cofactor biosynthesis protein NifE	*nifE*	blr1745	6.2	4.7	62.9	+	–
Bdiaspc4_08840	Nitrogenase iron-molybdenum cofactor biosynthesis protein NifN	*nifN*	blr1746	5.6	2.9	47.8	+	–
Bdiaspc4_08845	Nitrogen fixation protein NifX	*nifX*	blr1747	5.8	–	33.5	+	–
Bdiaspc4_08880	Hypothetical protein	–	bll1754	6.2	5.5	120.9	–	–
Bdiaspc4_08885	Iron-sulfur cluster assembly accessory protein	–	blr1755	5.0	7.0	138.7	+	–
Bdiaspc4_08890	Cysteine desulfurase NifS	*nifS*	blr1756	7.3	4.1	78.7	–	–
Bdiaspc4_08895	Putative nitrogen fixation protein NifT	*nifT*	bsr1757	5.6	4.8	105.4	–	–
Bdiaspc4_08905	Nitrogenase cofactor biosynthesis protein NifB	*nifB*	blr1759	5.8	2.9	74.1	+	–
Bdiaspc4_08945	OmpW family protein	–	bll1766	5.6	6.6	23.6	–	+
Bdiaspc4_08960	Nitrogenase iron protein	*nifH*	blr1769	7.9	7.0	96.2	+	–
Bdiaspc4_08975	Nitrogenase stabilizing/protective protein NifW	*nifW*	blr1771	5.4	2.2	95.2	–	–
Bdiaspc4_08985	Protein FixB	*fixB*	blr1773	6.0	2.2	47.6	–	–
Bdiaspc4_08990	Protein FixC	*fixC*	blr1774	5.8	–	38.1	–	–
Bdiaspc4_09875	Porin family protein	–	bll1944	8.4	3.8	89.7	+	–
Bdiaspc4_10340	SDR family oxidoreductase	*fixR*	blr2036	7.1	–	8.5	–	–
Bdiaspc4_10345	nif-specific transcriptional activator NifA	*nifA*	blr2037	6.6	–	3.2	–	–
Bdiaspc4_10350	Electron transfer flavoprotein subunit beta/FixAfamily protein	*fixA*	blr2038	5.9	–	31.9	+	–
Bdiaspc4_10455	Molecular chaperone GroEL	*groEL_*3*_*	bll2059	5.6	2.9	47.4	+	–
Bdiaspc4_10460	Co-chaperone GroES	*groES_*3*_*	bll2060	6.9	4.4	83.0	+	–
Bdiaspc4_10475	Flavin-dependent monooxygenase	*nrgC*	bll2063	5.2	4.4	105.0	+	–
Bdiaspc4_10495	Nodulate formation efficiency C protein	*nfeC*	bll2067	5.0	–	47.4	+	–
Bdiaspc4_10745	Non-ribosomal peptide synthetase	–	blr2108	6.5	–	39.0	+	–
Bdiaspc4_10870	Oxygenase	–	blr2131	5.5	15.9	298.2	–	–
Bdiaspc4_10935	Cytochrome P450	*cyp*112	blr2144	6.2	7.6	107.0	+	–
Bdiaspc4_10940	Cytochrome P450 BJ-3	*cyp*114	blr2145	6.5	3.3	55.0	+	–
Bdiaspc4_10950	Short-chain type dehydrogenase/reductase	–	blr2146	6.6	–	243.3	–	–
Bdiaspc4_10955	Cytochrome P450 BJ-4	*cyp*117	blr2147	6.2	2.4	26.4	–	–
Bdiaspc4_12590	Isocitrate lyase	*aceA*	blr2455	3.5	2.0	–	–	–
Bdiaspc4_13350	Universal stress protein	–	bll2590	3.1	11.1	5.2	–	–
Bdiaspc4_14280	Universal stress protein	–	blr2761	3.1	14.8	3.8	–	+
Bdiaspc4_14295	Cytochrome-*c* oxidase *cbb*_*3*_-type subunit II	*ccoO/fixO*	blr2764	3.9	47.0	26.8	–	+
Bdiaspc4_14305	*cbb*_3_-type cytochrome c oxidase subunit FixP	*fixP*	blr2766	5.6	43.7	23.9	–	+
Bdiaspc4_14950	1,2-phenylacetyl-CoA epoxidase subunit A	*ppaA*	blr2891	4.6	9.8	6.1	–	–
Bdiaspc4_18960	NAD(P)-dependent alcohol dehydrogenase	–	blr3675	6.4	–	54.4	–	–
Bdiaspc4_21300	Cation acetate symporter	–	blr4115	4.9	21.9	12.3	–	+
Bdiaspc4_21600	Glutamine synthetase 2	*glnII*	blr4169	2.0	–	2.1	–	–
Bdiaspc4_24320	Universal stress protein	–	bll4644	3.4	19.3	–	–	–
Bdiaspc4_24375	J domain-containing protein	*dnaJ*	blr4653	4.4	14.3	–	–	–
Bdiaspc4_24405	1-phosphofructokinase family hexose kinase	–	blr4659	5.2	12.1	–	–	–
Bdiaspc4_28005	Host attachment protein	–	bll5315	2.9	15.0	5.9	–	+
Bdiaspc4_31965	MCE family protein	–	bll6063	2.2	10.5	–	–	–
Bdiaspc4_31990	GNAT family N-acetyltransferase	–	bll6068	4.3	5.4	2.3	–	–
Bdiaspc4_32015	Alpha/beta fold hydrolase	*phaC2*	bll6073	3.3	13.5	4.1	–	+
Bdiaspc4_35215	Bacterioferritin	*bfr*	bll6680	1.7	–	–	–	–
Bdiaspc4_36320	Porin	–	bll6888	1.4	–	19.2	–	–
Bdiaspc4_36645	ModD protein	*modD*	bll6950	5.6	–	36.3	–	–
Bdiaspc4_36650	Molybdate ABC transporter substrate-binding protein	*modA*	blr6951	6.6	18.7	128.0	+	–
Bdiaspc4_37130	Periplasmic nitrate reductase subunit alpha	*napA*	blr7038	5.3	22.0	8.5	–	+
Bdiaspc4_38745	Hypothetical protein	–	blr7345	2.9	19.9	3.7	–	+
Bdiaspc4_39840	Hypothetical protein	–	bll7551	5.5	23.4	5.8	–	–
Bdiaspc4_41200	Hypothetical protein	–	bll7787	5.3	12.2	14.1	–	+
Bdiaspc4_41905	ABC transporter substrate-binding protein	–	bll7921	2.4	–	6.5	–	–
Bdiaspc4_41910	ABC transporter substrate-binding protein	–	blr7922	6.2	–	23.1	–	–
Bdiaspc4_42205	Dehydrogenase	–	bll7981	4.6	7.6	2.1	–	+
Bdiaspc4_42210	Class I SAM-dependent methyltransferase	–	bll7982	3.7	10.5	3.6	–	+
**Four proteins showing decreased expression in microoxic conditions in comparison to oxic conditions (*****p*****-value** **≤** **0.2)**								
Bdiaspc4_01330	PQQ-dependent dehydrogenase, methanol/ethanol family	–	bll0333	−0.8	–	–	–	–
Bdiaspc4_05515	Sugar ABC transporter substrate-binding protein	–	blr1123	−3.0	–	–	–	–
Bdiaspc4_36200	Flagellin	*fliC/lafA2*	bll6865	−1.8	–	–	–	–
Bdiaspc4_39525	GMC Family oxidoreductase	–	blr7491	−2.3	–	–	–	–
**Seventy-one genes/proteins showing increased expression in microoxic conditions in comparison to oxic conditions (log**_**2**_ **FC** **≥** **1) at both trancriptional and protein levels not included above**								
Bdiaspc4_01235	TAT-dependent nitrous-oxide reductase	*nosZ*	blr0315	1.8	2.4	–	–	–
Bdiaspc4_02185	Hypothetical protein	–	blr0497	3.8	14.1	12.7	–	+
Bdiaspc4_02350	SDR family oxidoreductase	–	bll0527	1.1	2.9	–	–	–
Bdiaspc4_06385	Long-chain fatty acid-CoA ligase	–	blr1288	3.2	3.9	3.6	–	–
Bdiaspc4_06390	Oleate hydratase	–	blr1289	3.2	19.0	2.7	–	+
Bdiaspc4_06420	Aldo/keto reductase	–	bll1295	1.2	3.0	–	–	–
Bdiaspc4_06685	Hypothetical protein	–	bll1342	1.7	2.5	–	–	–
Bdiaspc4_06740	Amidohydrolase	–	blr1352	1.7	2.1	–	–	–
Bdiaspc4_07460	Sulfate ABC transporter substrate-binding protein	–	blr1482	1.2	3.2	–	–	–
Bdiaspc4_08965	Nitrogen fixation protein NifQ	*nifQ*	blr1770	4.6	3.2	85.0	–	–
Bdiaspc4_09000	Alkyl hydroperoxide reductase AhpD	*ahpD*	bll1776	4.7	4.1	78.6	+	–
Bdiaspc4_09005	Peroxiredoxin	*ahpC*	bll1777	4.5	7.8	169.8	+	–
Bdiaspc4_09500	Omptin family outer membrane protease	–	bll1872	4.7	4.2	115.4	+	–
Bdiaspc4_09550	RNA polymerase σ^54^ factor	*rpoN_*1*_*	blr1883	4.4	3.9	7.8	–	+
Bdiaspc4_09680	GNAT family N-acetyltransferase	–	bll1906	4.3	8.3	120.0	+	–
Bdiaspc4_10185	Oxygen-independent coproporphyrinogen III oxidase	*hemN_*1*_*	bll2007	4.5	17.3	29.1	–	+
Bdiaspc4_10960	Polyprenyl synthetase family protein	–	blr2148	4.6	4.2	25.4	–	–
Bdiaspc4_11365	Aspartate aminotransferase family protein	–	blr2221	1.5	2.9	–	–	–
Bdiaspc4_12935	Phosphoketolase family protein	–	bll2518	4.3	8.2	–	–	–
Bdiaspc4_14265	Response regulator	–	bll2758	4.8	5.5	2.4	–	–
Bdiaspc4_14285	CBS domain-containing protein	–	blr2762	4.8	13.2	3.1	–	–
Bdiaspc4_14310	Cytochrome c oxidase accessory protein CcoG	*ccoG/fixG*	blr2767	4.3	32.5	13.0	–	+
Bdiaspc4_14320	Copper-translocating P-type ATPase	*fixI*	blr2769	3.8	23.5	11.0	–	+
Bdiaspc4_14930	Phasin	–	blr2887	1.8	2.4	3.4	–	–
Bdiaspc4_14960	Phenylacetate-CoA oxygenase subunit PaaI	*paaI*	blr2893	2.1	9.3	5.6	–	–
Bdiaspc4_14970	Phenylacetate-CoA oxygenase/reductase subunit PaaK	*paaK*	blr2895	1.9	5.0	2.6	–	–
Bdiaspc4_14980	Phenylacetate-CoA ligase	*paaF*	blr2897	2.0	4.5	–	–	–
Bdiaspc4_16100	MBL fold metallo-hydrolase	–	bll3115	2.9	15.4	–	–	–
Bdiaspc4_16105	Ribose-phosphate pyrophosphokinase	–	bll3116	4.4	7.7	–	–	–
Bdiaspc4_16110	Thymidine phosphorylase family protein	–	bll3117	2.1	5.1	–	–	–
Bdiaspc4_19720	HAD family hydrolase	–	blr3815	3.7	5.8	–	–	–
Bdiaspc4_20685	NAD-dependent succinate-semialdehyde dehydrogenase	–	bll3998	2.6	28.1	8.4	–	+
Bdiaspc4_21285	CusA/CzcA family heavy metal efflux RND transporter	–	blr4112	4.8	6.4	–	–	–
Bdiaspc4_21700	Hypothetical protein	–	bsl4187	4.5	2.3	–	–	–
Bdiaspc4_22005	Pyridoxamine 5'-phosphate oxidase family protein	–	blr4240	3.8	12.5	–	–	–
Bdiaspc4_22345	Amidase	–	bll4303	1.6	3.9	–	–	–
Bdiaspc4_22980	Translational machinery protein	–	bll4412	2.6	6.6	–	–	–
Bdiaspc4_24325	Host attachment protein	–	bll4645	3.2	21.8	–	–	–
Bdiaspc4_24330	CBS domain-containing protein	–	blr4646	3.2	28.1	–	–	–
Bdiaspc4_24365	Protein-L-isoaspartate(D-aspartate) O-methyltransferase	–	bll4651	4.6	28.7	9.7	–	+
Bdiaspc4_24370	Nitroreductase	–	blr4652	2.9	51.6	5.9	–	+
Bdiaspc4_24385	Phosphoenolpyruvate synthase	–	blr4655	4.3	11.2	2.0	–	–
Bdiaspc4_24400	Glucokinase	*glk*	blr4658	3.7	9.4	2.9	–	–
Bdiaspc4_27515	Co-chaperone GroES	*groES_*1*_*	blr5226	3.4	2.1	–	–	–
Bdiaspc4_29305	CBS domain-containing protein	–	bll5551	1.1	2.0	–	–	–
Bdiaspc4_29865	Alcohol dehydrogenase AdhP	*adhP*	bll5655	2.8	18.0	3.9	–	+
Bdiaspc4_30480	Efflux RND transporter permease subunit	–	bll5771	3.8	2.6	–	–	–
Bdiaspc4_30485	Efflux RND transporter periplasmic adaptor subunit	–	bll5772	4.0	3.5	–	–	–
Bdiaspc4_30510	DUF302 domain-containing protein	–	blr5777	4.3	5.0	3.3	–	–
Bdiaspc4_30515	Cytochrome c oxidase accessory protein CcoG	*ccoG/fixG*	blr5778	3.4	4.6	4.1	–	–
Bdiaspc4_31945	Cyclase family protein	–	blr6059	1.5	2.6	–	–	–
Bdiaspc4_31950	CRP/FNR family transcriptional regulator	–	bll6060	1.3	2.3	2.2	–	–
Bdiaspc4_31970	ABC transporter ATP-binding protein	–	bll6064	2.8	4.6	2.1	–	–
Bdiaspc4_31975	MlaE family lipid ABC transporter permease subunit	–	bll6065	3.4	6.7	–	–	–
Bdiaspc4_31995	Universal stress protein	–	bll6069	2.7	18.5	7.5	–	+
Bdiaspc4_32020	CBS domain-containing protein	–	blr6074	2.6	35.6	4.7	–	+
Bdiaspc4_34005	Methyltransferase	–	bll6449	1.4	2.7	–	–	–
Bdiaspc4_34035	ABC transporter substrate-binding protein	–	bll6455	2.8	4.4	–	–	–
Bdiaspc4_34375	Aryl-sulfate sulfotransferase	–	bsr6521	2.6	3.4	–	–	–
Bdiaspc4_36795	Chaperonin GroEL	*groEL_*2*_*	blr6979	1.6	3.9	2.0	–	–
Bdiaspc4_37135	Nitrate reductase cytochrome c-type subunit	*napB*	blr7039	2.8	47.4	22.6	–	–
Bdiaspc4_37380	CRP/FNR family transcriptional regulator	*nnrR*	blr7084	3.6	7.1	–	–	–
Bdiaspc4_37390	Oxygen-independent coproporphyrinogen III oxidase	*hemN_*2*_*	bll7086	4.3	29.4	7.3	–	+
Bdiaspc4_37770	DUF2852 domain-containing protein	–	bll7160	2.5	2.1	4.9	–	–
Bdiaspc4_39110	Elongation factor G	–	bll7414	1.9	4.1	2.8	–	–
Bdiaspc4_40040	Indolepyruvate ferredoxin oxidoreductase family protein	–	blr7589	1.1	3.6	–	–	–
Bdiaspc4_40955	Molecular chaperone	–	blr7740	3.2	2.1	4.5	–	–
Bdiaspc4_41165	Hypothetical protein	–	blr7780	4.5	8.6	17.0	–	–
Bdiaspc4_41645	HlyD family efflux transporter periplasmic adaptor subunit	–	blr7872	3.5	14.1	–	–	–
Bdiaspc4_42105	Hsp20/alpha crystallin family protein	–	blr7961	4.8	28.4	25.2	–	+
Bdiaspc4_43115	HugZ family protein	–	bll8143	3.2	2.2	–	–	–

Shown are also 71 genes/proteins; i.e., 117 genes/proteins comprising the “expanded microoxia-induced transcriptome/proteome” (log_2_ fold change ≥ 1 at both transcriptional and protein level) minus 46 genes/proteins included in the “core microoxic proteome.”

a *Nomenclature of B. diazoefficiens 110spc4 genes according to the NCBI annotation (GenBank acc. # CP032617); this work*.

b Protein/gene product according to the NCBI annotation (GenBank acc. # CP032617); this work.

c Gene name according to the NCBI annotation with modifications shaded in gray (GenBank acc. # CP032617); this work.

d Best blast hit in the B. diazoefficiens USDA 110 genome (Kaneko et al., 2002; GenBank acc. # NC_004463.1; RefSeq annotation as from January 2016).

e Log_2_ fold change (FC) values from the comparison of wild-type cells grown microoxically (0.5% O_2_) in comparison with those of cells grown oxically in proteomics experiments.

f FC values from the comparison of wild-type cells grown microoxically (0.5% O_2_) in comparison with those of cells grown oxically in transcriptomics experiments (Pessi et al., 2007). – indicates the gene was not differentially expressed.

g FC values from the comparison of wild-type soybean bacteroids in comparison with those of wild-type cells grown oxically in transcriptomics experiments (Pessi et al., 2007). – indicates the gene was not differentially expressed.

h The gene belongs to the defined NifA + RpoN_1+2_ regulon according to Hauser et al. (2007). +, yes; –, not.

i *The gene is part of the direct FixK_2_-dependent regulon as defined by Mesa et al. (2008). +, yes; –, not*.

To further explore the overlap of microoxically induced proteins with the microoxia-induced transcriptome of 603 protein-coding genes (fold change ≥ 2; i.e., log_2_ FC ≥ 1) (Pessi et al., 2007), we went beyond the top statistically differentially regulated proteins identified by DESeq2 and now also considered proteins upregulated by a log_2_ FC of more than 1 in microoxia. Applying this more lenient threshold returned 206 microoxia-induced proteins (Supplementary Table S3, Data Sheet F); their overlap with the 603 protein-coding genes amounted to 117 genes/proteins that form the “expanded microoxia-induced transcriptome/proteome” (i.e., 71 extra genes/proteins in addition to the group of 46 that overlap with the “core microoxic proteome”; Figure 4B; Table 2). This group of 71 genes/proteins includes four NifA+RpoN_1+2_ targets, 14 FixK_2_ direct targets, and 38 genes induced at transcriptional level in wild-type soybean bacteroids in comparison to oxic conditions (Pessi et al., 2007). Overall, the latter comparison showed an overlap of 66% (78 out of 117 microoxia-induced genes/proteins) (Table 2).

### Microoxia-Mediated Post-transcriptional Regulation

**Figure 5:**
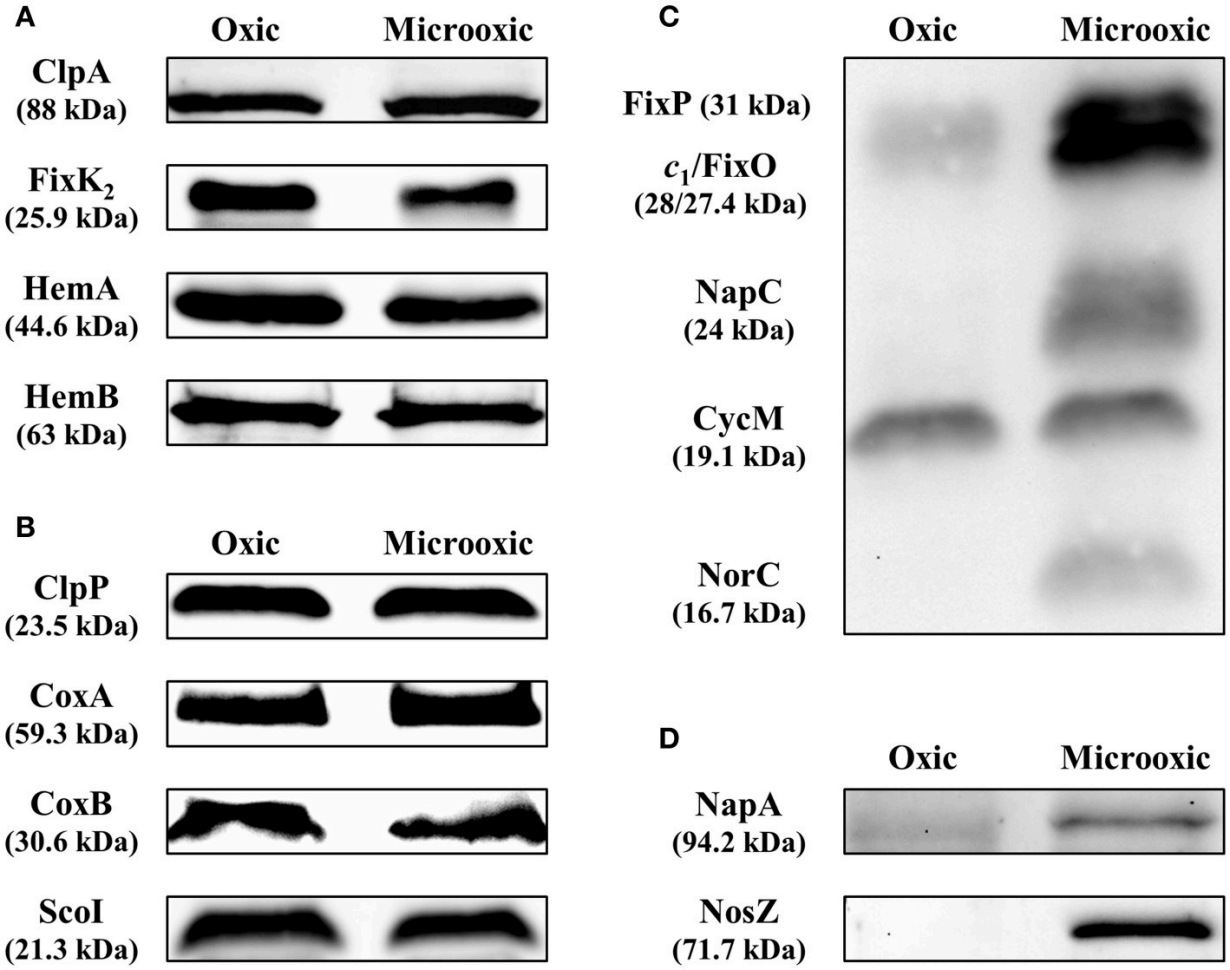
Validation of protein abundance for genes subject to post-transcriptional regulation determined by heme-staining and western blot analyses. Steady-state levels of ClpA, FixK_2_, HemA, and HemB **(A)**, targets subject to post-transcriptional control, were analyzed by western blot. ClpP, CoxA, CoxB, and ScoI, proteins with constant accumulated levels, were included as controls **(B)**. Up-regulation of the membrane-bound FixO and FixP proteins and the periplasmic, soluble NapA and NosZ proteins were monitored by heme-staining **(C)** or western blot **(D)**. The membrane-bound cytochrome CycM was used as reference in the heme-staining experiments in **(C)** as the protein levels remained constant in both oxic and microoxic conditions. Crude extracts, soluble and membrane fractions were isolated from *B. diazoefficiens* cells cultivated oxically and microoxically. 10–40 μg of cytosolic (ClpA, ClpP, HemA, HemB, NapA, NosZ), membrane (CoxB, ScoI), or crude extract (CoxA, FixK_2_) fractions were loaded in the gel in the western blot experiments (identical amount of protein extracted from cells grown oxically and microoxically for each validated target). Twenty-five microgram membranes were loaded in the gel for the heme-staining analyses. Apparent molecular masses of the proteins are shown on the left. Note that as described previously (Loferer et al., 1993), the CoxA protein migrated at ≈45 kDa instead of the corresponding predicted mass of 59.3 kDa. Shown are representative results of different experiments carried out with at least three independent biological replicates. A detailed description of the methodology is described in Supplementary Table S5.

The integration of our proteomics data with previous transcriptomics data revealed that for 486 protein-coding genes induced under microoxic conditions (603 minus 117; Figure 4B), there was only a more moderate or even no correlation between transcript and protein abundance. Indeed, in contrast to the induction of the respective genes under microoxia, a group of 91 proteins was even downregulated (log_2_ FC ≤ 0.5 or *p*-value ≥ 0.9) under microoxic conditions compared to oxic conditions (Supplementary Table S4). Interestingly, such a profile was previously observed for the *fixK*_2_ gene which encodes the transcription factor FixK_2_ that plays a key role in microoxia-mediated regulation. Transcriptional induction of the *fixK*_2_ gene in cells grown under microoxic conditions in comparison to oxic conditions (Nellen-Anthamatten et al., 1998; Pessi et al., 2007) did not correlate with increased steady-state levels of the FixK_2_ protein (Mesa et al., 2009). To identify potential target genes with a regulation similar to that described for *fixK*_2_, we applied the following selection criteria for the list of 91 genes/proteins: (i) Soluble proteins (using the combined prediction of potential protein localization, TM helices, and signal peptides as described in the Materials and Methods section). (ii) Genes that code for proteins with an assigned function according to the NCBI annotation (GenBank accession number CP032617). (iii) Availability of antibodies against the corresponding *B. diazoefficiens* protein or a cross-reacting protein ortholog. While 59 targets fulfilled criteria (i) and (ii), only three proteins also fulfilled criterion (iii) (Supplementary Table S4). These three targets were selected for validation by western blot analysis, along with the FixK_2_ protein (Bdiaspc4_14260 gene) as positive control (Figure 5A): HemA (Bdiaspc4_05915 gene) coding for a 5-aminolevulinate (ALA) synthase (Page and Guerinot, 1995; Jung et al., 2004), HemB (Bdiaspc4_26465 gene) coding for a porphobilinogen synthase or ALA dehydratase (Chauhan and O'Brian, 1995), and ClpA (Bdiaspc4_27090) coding for the ATP-binding chaperone component of the ClpAP proteolytic system (Bonnet et al., 2013). As further controls, we also included four genes not induced in microoxia (Pessi et al., 2007), whose products were accumulated to constant levels (Figure 5B): ClpP (Bdiaspc4_25960 gene) categorized as a soluble protein, CoxA or CtaD (Bdiaspc4_05770 gene), CoxB (Bdiaspc4_05765 gene), and ScoI (Bdiaspc4_05560 gene) classified as membrane proteins. Crude extracts, soluble and membrane fractions were isolated from *B. diazoefficiens* cells cultivated oxically and microoxically, separated by SDS-PAGE, and immunoblotted against the corresponding antibody (a list of antibodies used in this work together with associated details of the immunoblot methodology are listed in Supplementary Table S5). Similar to the constant accumulated levels of ClpP_1_, CoxA, CoxB, and ScoI, steady-state levels of ClpA, FixK_2_, HemA, and HemB were comparable in cells of *B. diazoefficiens* cultivated oxically or microoxically (Figure 5A), despite of the induction of the respective genes in microoxic conditions (Supplementary Table S4; Pessi et al., 2007). Together, these data suggested that in addition to *fixK*_2_ other genes might also be under microoxia-mediated post-transcriptional control in *B. diazoefficiens*.

We also validated the up-regulation of the membrane proteins FixO and FixP, and the periplasmic proteins NapA and NosZ, respectively, by heme-staining (Figure 5C) and immunoblotting (Figure 5D), using the same membrane and cytosolic samples employed in Figures 5A,B. The heme-staining technique allows detection of proteins covalently bound to heme (Vargas et al., 1993) such as cytochromes. In line with the differential protein abundance (see above), the protein bands corresponding to FixO, and FixP were more prominent in membrane preparations from the wild-type strain grown microoxically, while the accumulated levels of CycM, a cytochrome of about 19 kDa encoded by the Bdiaspc4_07150 gene, remained constant irrespective of the growth condition (Figure 5C). Similarly, we validated the upregulation of NapA and NosZ proteins by using heterologous antibodies (see Materials and Methods) (Figure 5D).

## Discussion

We applied an integrated systems biology approach to analyze the adaptation of a key rhizobial model species to microoxic conditions. We used *B. diazoefficiens* strain 110*spc*4 as a model, as a wealth of functional genomics data have been compiled for this strain. These comprise microarray-based gene expression and RNA-Seq studies, including experiments carried out under microoxia, a number of shotgun proteomics studies and efforts to integrate transcriptomics, proteomics and metabolomics datasets (Hauser et al., 2007; Lindemann et al., 2007; Pessi et al., 2007; Hacker et al., 2008; Lang et al., 2008; Mesa et al., 2008, 2009; Delmotte et al., 2010; Koch et al., 2010, 2014; Reutimann et al., 2010; Masloboeva et al., 2012; Serventi et al., 2012; Torres et al., 2014; Čuklina et al., 2016; Lardi et al., 2016; reviewed in Lardi and Pessi, 2018). Motivated by previous observations that the genome of this important strain might differ from that of the NCBI USDA 110 reference strain (Kaneko et al., 2002), we sequenced and *de novo* assembled the complete genome of *B. diazoefficiens* 110*spc*4 using long reads from PacBio's third generation sequencing technology. As predicted by a repeat complexity analysis of close to 10'000 prokaryotic genomes (Schmid et al., 2018), this approach was straightforward as *B. diazoefficiens* genomes do not harbor very long repeats that complicate genome assembly. Furthermore, a complete genome sequence is the basis to identify differences compared to a prior reference genome sequence using proteogenomics: such differences can affect annotated genes as shown in Supplementary Figure S5A, where proteogenomic evidence of a peptide directly confirms the protein sequence of a CDS in strain 110*spc*4 that is affected by a frameshift in USDA 110. Moreover, proteogenomics can also identify strain-specific and/or previously unannotated short protein-coding genes (Omasits et al., 2017), evidence for expressed pseudogenes (Supplementary Figure S5B), and even provide evidence for shorter proteins than annotated (Čuklina et al., 2016). Corroborating earlier hints (Mesa et al., 2001), we indeed identified a genomic deletion of roughly 202 kb in strain 110*spc*4, which however did not show any significantly different symbiotic phenotype compared to strain USDA 110. The extensive table with the genomic coordinates of all protein-coding genes of *B. diazoefficiens* 110*spc*4, the respective homolog of strain USDA 110, additional functional predictions and all experimental evidence (Supplementary Table S3, Data Sheet A) will facilitate researchers to readily compare datasets that were using the USDA 110 annotation.

The results of our proteomics analysis provide the first extensive protein expression dataset for microoxic growth of *B. diazoefficiens*. We deliberately selected the FASP method, which is known to achieve a higher coverage of membrane proteins (Wiśniewski et al., 2009a,b), that otherwise are typically significantly underrepresented in shotgun proteomics studies (Ahrens et al., 2010). Indeed, compared to the study of Delmotte et al. (2010), where ~7.8% of the 2,315 experimentally identified root nodule proteins were classified as TM proteins, we identified close to 12% of TM proteins in our dataset. This percentage is still lower than that of the entire 110*spc*4 proteome (19.9%); however, we did not apply the costly and time-consuming iterative feedback loop strategy that was used in one of the only studies that accomplished a complete membrane proteome coverage to date (Omasits et al., 2013).

Applying an adjusted *p*-value ≤ 0.2 as first cut-off, 62 proteins were significantly upregulated (Figure 4A; Table 2), including many targets known to be important for the microoxic free-living and symbiotic lifestyles, including NifD, NifK, NifH, FixO, FixP, NifA, PhaC2, and AcdS. These results reinforced our previous findings, that the conditions in our experiments mimic the microoxic environment inside root nodules. Likewise, using this cut-off, four proteins were significantly downregulated in cells grown under microoxic conditions. Interestingly, one of those is FliC (Bdiaspc4_36200, bll6865 in USDA 110), a protein that plays a role in the lateral flagellar system in *B. diazoefficiens*, which was named LafA2 (Mongiardini et al., 2017). Bacterial motility is seemingly beneficial for a free-living organism in the environment, where the lateral system contributes to swimming in wet soil and competition for nodulation. For instance, a mutant lacking lateral flagellar filaments was found to be more competitive for nodulation than the wild type (Althabegoiti et al., 2011). Furthermore, expression of the *fliC*/*lafA2* gene was upregulated in oxic conditions (Pessi et al., 2007) and was shown to be under the positive control of the RegR regulatory protein (Lindemann et al., 2007).

Applying a more relaxed selection threshold (log_2_ FC ≥ 1; i.e., the same as used in the prior transcriptomics study) a total of 117 genes/proteins represented the “expanded microoxia-induced transcriptome/proteome” (Table 2). Among these proteins/genes upregulated in microoxia we found: (i) The denitrifying proteins NapA, NapB (subunits of the periplasmic nitrate reductase) and NosZ, the structural subunit of the nitrous oxide reductase (Delgado et al., 2003; Velasco et al., 2004). It is worth mentioning that microoxia has been addressed as the key signal for the expression of nitrate-, nitrite- and nitrous oxide reductase-encoding genes in the denitrification process in *B. diazoefficiens* (Bueno et al., 2017; Torres et al., 2017). (ii) Two copies of the oxygen-independent coproporphyrinogen III oxidase involved in heme biosynthesis under low-oxygen conditions (i.e., HemN_1_ and HemN_2_). Mutant strains with a defective *hemN*_2_ gene failed to grow under denitrifying conditions and were also unable to fix nitrogen in symbiosis with soybeans (Fischer et al., 2001). (iii) Subunits of the FixGHIS complex, i.e., CcoG/FixG (Bdiaspc4_14310) and FixI (Bdiaspc4_14320) required for the functional assembly of the high affinity *cbb*_3_ terminal oxidase FixNOQP (Preisig et al., 1996). (iv) The AhpD and AhpC proteins that form part of an alkylhydroperoxide reductase (Ahp), whose encoding genes were reported to be upregulated in soybean bacteroids and also belong to the NifA + RpoN_1+2_ regulon (Hauser et al., 2007; Pessi et al., 2007). Although the Ahp reductase might play a role in the bacterial response against reactive oxygen species produced during rhizobia-legumes interaction (Pauly et al., 2006; Damiani et al., 2016), its exact role in the microoxic metabolism of this bacterium is under debate. (v) The product of the Bdiaspc4_20685 gene (bll3998 in USDA 110), a succinate-semialdehyde dehydrogenase that probably acts a tricarboxylic acid cycle bypass enzyme (Green et al., 2000). Expression of bll3998 was activated by FixK_2_ and also found to be upregulated in soybean bacteroids (Pessi et al., 2007; Mesa et al., 2008). (vi) The glyoxylate shunt enzyme isocitrate lyase (AceA) which also plays a protective role in the response to several stresses (Jeon et al., 2015). (vii) The subunits ModA (Bdiaspc4_36650) and ModD (Bdiaspc4_36645) that form part of one of the predicted molybdenum transport systems (Kaneko et al., 2002; Delgado et al., 2006). Molybdenum is the cofactor of the oxygen-sensitive enzyme nitrogenase and the periplasmic nitrate oxide reductase. Expression of *modA* was highly induced in soybean bacteroids and it also belongs to the NifA + RpoN_1+2_ regulon. (viii) Several regulatory proteins such as the CRP/FNR-type transcription factor NnrR involved in denitrification (Mesa et al., 2003) or the alternative sigma factor RpoN_1_.

Among the microoxia-induced proteins that did not correlate with increased transcript levels of the corresponding gene we found two proteins involved in iron metabolism, i.e., a bacterioferritin (encoded by Bdiaspc4_35215, bll6680 in USDA 110; Table 2; Supplementary Table S3, Data Sheet F) and a rubrerythrin (encoded by Bdiaspc4_41765, blr7895 in USDA 110; Supplementary Table S3, Data Sheet F). The expression of these two genes was induced in iron-replete media (Rudolph et al., 2006), in agreement with their assigned function to maintain proper levels of free iron in the cells. As they share oxygen-sensitive structural features (deMaré et al., 1996), they might be degraded in oxic conditions. Finally, we also identified a set of 639 genes/proteins that were only expressed in microoxia (Supplementary Table S3, Data Sheet E).

Our data set greatly increased the proteome coverage compared to previous 2-D gel-based studies (Regensburger et al., 1986; Dainese-Hatt et al., 1999). The more recent of the studies reported 38 differentially regulated proteins when comparing anoxic and low oxygen (2% O_2_) conditions to oxic conditions, 34 of which were also controlled by either NifA or FixK_2_. Fourteen of these proteins could not be identified by N-terminal sequencing, peptide mass fingerprinting and MS/MS analysis at the time. When searching the respective *N*-terminal protein sequences reported in the original paper (Dainese-Hatt et al., 1999) against our *de novo* assembled genome sequence, we could readily identify several of them. These included Bdiaspc4_24330 (blr4646 in USDA 110, CBS domain-containing protein; spot 17.1; Table 2; Supplementary Table S3, Data Sheet F) and Bdiasspc4_09275 (blr1830 in USDA 110, hypothetical protein; spot 5.1; Supplementary Table S3, Data Sheet F), both of which were also among the upregulated proteins under microoxic conditions in our study (0.5% O_2_).

More recently, RNA-Seq studies performed in rhizobial species grown under low-oxygen conditions (Schlüter et al., 2013; Robledo et al., 2015; Čuklina et al., 2016) revealed a poorly characterized post-transcriptional control via ubiquitous small RNA-dependent regulation. Here we focus on post-transcriptional control of protein-encoding genes that are regulated in response to the cellular oxygen status. Differences between the patterns of accumulated transcripts and proteins could be due to condition-dependent differences in transcript and protein stabilities or stochastic effects of protein synthesis from scarce and unstable mRNA molecules (Taniguchi et al., 2010, and references therein). Our study identified a group of genes whose transcript and protein profile is similar to the one described for the *fixK*_2_ gene, and therefore probably subject to microoxia-specific post-transcriptional control: Despite transcriptional induction in response to microoxic conditions there is no increase in the amount of the protein (Nellen-Anthamatten et al., 1998; Mesa et al., 2008). The FixK_2_ protein is also post-translationally controlled by oxidation (Mesa et al., 2009) and by proteolysis mediated by the ClpAP proteolytic system (Bonnet et al., 2013). In order to validate other potential genes subjected to post-transcriptional regulation, we performed western blot analyses with antibodies available against the proteins encoded by three genes: *hemA, hemB*, and *clpA*.

The 5-aminolevulinate (ALA) synthase (EC:2.3.1.37) HemA and the ALA dehydratase (EC:4.2.1.24) HemB catalyze the first two steps of heme biosynthesis in *B. diazoefficiens*. In addition to iron control of *hemA* and *hemB* expression *via* two different regulatory mechanisms (Hamza et al., 2000; reviewed in O'Brian, 2015), Page and Guerinot (1995) reported that *hemA* expression is also under post-transcriptional regulation, as increased *hemA* mRNA levels in bacteroids compared to free-living cells were not accompanied by elevated ALA synthase activity. The third validated candidate under putative post-transcriptional control is *clpA* encoding the ATP-dependent chaperone component of the ClpAP proteolytic system involved in FixK_2_ degradation (Bonnet et al., 2013). ClpA itself is also a specific substrate for the ClpAP system with the recognition signal located within the C-terminal 12 amino acids. Notably, the recognition sequence of *E. coli* ClpA is also C-terminally located since a protein variant lacking the last nine residues is protected from auto-degradation (Maglica et al., 2008). Furthermore, post-translational control of ClpA is not dependent on oxygen limitation. Here, we confirmed previous observations on the absence of other FixK paralogs in the nodule proteome determined by Delmotte et al. (2010). In fact, the other three predicted *B. diazoefficiens* FixK paralogs (i.e., Bll2109, [Bdiaspc4_10755 gene]; Bll3466, [Bdiaspc4_17910 gene]; Bll7696, [Bdiaspc4_40670 gene]; (Mesa et al., 2006) were not detected at the protein level under microoxic conditions despite the respective genes belong to the group of 603 protein-coding genes with significantly increased transcript levels in microoxia (Pessi et al., 2007).

Altogether our data suggest that additional genes beyond *fixK*_2_ are subject to microoxia-mediated post-transcriptional control in *B. diazoefficiens*. A more detailed analysis of this type of regulation and elucidating its physiological relevance are interesting avenues for future investigations.

## Data Availability

The genome sequence of *B. diazoefficiens* 110*spc*4 is available from Genbank under accession CP032617 (BioProject: PRJNA493766). In addition, the raw sequence data (and methylation analysis) has been submitted to the NCBI Sequence Read Archive (SRA) which can be accessed under study number: SRP162984. The mass spectrometry proteomics data have been deposited to the ProteomeXchange Consortium via the PRIDE (Perez-Riverol et al., 2019) partner repository with the dataset identifier PXD012491 and 10.6019/PXD012491. An integrated proteogenomics database (iPtgxDB) for *B. diazoefficiens* 110*spc*4 will be available (https://iptgxdb.expasy.org).

## Author Contributions

NF performed the proteomics experiments and contributed to data interpretation and manuscript writing. AV performed the genome comparison, proteogenomics analysis, analyzed proteomics data, contributed to the comparison with prior transcriptomics data and manuscript writing, and prepared figures and tables. JC performed the western blot and heme-staining experiments, contributed to data interpretation, and prepared figures and tables. SL performed the *de novo* genome assembly, genome comparison, and contributed to figures and tables. BR also performed proteomics experiments. RL and H-MF contributed genomic DNA of strain 110*spc*4, data interpretation, and compared the symbiotic phenotype of strains USDA 110 and 110*spc*4 in symbiosis with different plant hosts. LE and EB critically revised the manuscript. CA oversaw sequencing, *de novo* genome assembly and comparison, data analysis and proteogenomics. GP devised the proteomics shotgun strategy, and contributed to the study design, and data interpretation. SM conceived and designed the study, contributed to the comparison with previous transcriptomics data, devised the strategy for selection of post-transcriptionally regulated targets, and interpreted data. CA, GP, and SM wrote the manuscript. All authors read and approved the final version of the manuscript.

### Conflict of Interest Statement

The authors declare that the research was conducted in the absence of any commercial or financial relationships that could be construed as a potential conflict of interest. The handling editor declared a past supervisory role with one of the authors RL.

## Supplementary Material

Supplementary Figure S1 PCR verification of the ~202 kb deletion present in *Bradyrhizobium diazoefficiens* USDA 110 derivatives 110*spc*4 (Regensburger and Hennecke, 1983) and 110*rif* 15 (Regensburger and Hennecke, 1984). **(A)** Schematic representation of the genomic region comprising the deletion between genome position no. 8,974,768 and 70,634 in the reference strain USDA 110 (acc. no. NC_004463.1). Primer binding sites and corresponding amplification products are indicated by horizontal arrows and thin solid lines, respectively. Not drawn to scale. **(B)** Analysis of PCR products obtained with indicated primer combinations and genomic template DNA from different *B. diazoefficiens* strains. No PCR products were obtained with strain 110*spc*4 when at least one primer binding site was located within the deleted region (primer combinations Del-1/2, 3/4, 5/6). By contrast, a 1,031 bp product spanning the deletion was obtained when primers Del-2/3 were used. No differences between the resequenced clone (a; preserved in 2018) and the oldest available clone (b; preserved in 1987) was observed. Identical PCR results were obtained with strain 110*rif* 15, which was independently isolated from the same parental strain as 110*spc*4. Using genomic DNA of strain USDA 110 as template, amplification products spanning the ends of the 203 kb region (Del-1/2, 3/4) and an internal fragment thereof (Del-5/6) were obtained. The very weak band in the outermost right lane likely represents an off-target product amplified by primers Del-2/-3 from USDA 110 template DNA.

Supplementary Figure S2 Leaf phenotype of different legume hosts inoculated with *Bradyrhizobium diazoefficiens* 110*spc*4 or USDA 110. All tested host plants [*Glycine max* cv. Williams 82 and cv. Black jet (soybeans), *Vigna radiata* (mung bean), *Vigna unguiculata* (cowpea), *Macroptillium atropurpureum* (siratro), and *Aeschynomene afraspera*] displayed dark-green leaves when inoculated with either 110*spc*4 (center) or USDA 110 (right) compared to the chlorotic, yellowish leaves of non-inoculated plants (left) typical for nitrogen-starved plants. No qualitative differences between 110*spc*4- and USDA 110-inoculated plants were visible. Shown are three leaves of three representative plants for each condition. The necrotic spots on *V. radiata* leaves regularly appear under the given growth conditions, independently of the inoculated strain and do not interfere with symbiosis.

Supplementary Figure S3 Quality assessment of the shotgun proteomics samples (3 biological replicates each for growth under oxic and microoxic conditions) by a clustering analysis (heatmap based on semi-quantitative spectral count data).

Supplementary Figure S4 Venn diagram showing the overlap of 110*spc*4 orthologs (see section Materials and Methods) of the 506 previously identified microoxia-specific transcribed genes (Pessi et al., 2007) with the 288 proteins uniquely expressed in microoxia (this study) and 2,900 proteins expressed in oxia (this study). The overlap revealed that 647 genes/proteins were only expressed in microoxia (506-75-72+288). Among these 647, six genes are not present (annotated) in the *B. diazoefficiens* 110*spc*4 genome, one is affected by a frameshift (blr6071) and one gene is encoded in the 202 kb deletion region (bsr0039; erroneously implicated to be transcribed likely due to cross-hybridization with cDNA originating from another gene) resulting in 639 microoxia-specific genes/proteins (Supplementary Table S3, Data Sheet E).

Supplementary Figure S5 Example of proteogenomic evidence for novelties based on genome sequence differences. **(A)** The correct genomic sequence of strain 110*spc*4 is shown for a region of interest along with that of the USDA 110 reference strain and the respective annotations. Bdiaspc4_16655 is annotated as a sugar ABC transporter gene in strain 110*spc*4 (upper panel), predicted to encode a protein of 438 amino acids (blue arrow). A single nucleotide deletion in the genome sequence of strain USDA110 causes a frameshift. Accordingly, two shorter protein-coding genes are annotated (blr3220, blr3221; gray arrows) in the same reading frame. The peptide evidence underlines that our *de novo* genome assembly is correct: we observed a peptide (red peptide on the left) whose sequence directly confirmed the change compared to USDA 110 and another one traversing the incorrect stop codon (adjacent red peptide). **(B)** Additional examples can be uncovered using the publicly available iPtgxDB for strain 110*spc*4. Here, a genomic region of 110*spc*4 is shown where the NCBI annotation listed two CDSs (Bdiaspc4_31490, Bdiaspc4_31500; blue arrows) and one pseudogene (Bdiaspc4_31495; open arrow). We find proteogenomic evidence for this ORF (red peptides), which was predicted to represent a *bona fide* CDS by Prodigal (gray boxes; particular gene identifier highlighted in red), underlining the value of such iPtgxDBs to improve the genome annotation of prokaryotic genomes (Omasits et al., 2017).

Supplementary Data Sheet 1 List of references included in the Supplementary Material.

Supplementary Table S1 List of 223 CDS located in the ~202 kb genomic region that is deleted in *B. diazoefficiens* 110*spc*4 compared to the USDA110 NCBI reference genome (corresponding to nucleotide positions 8,974,768–9,105,828 and 1–70,634). The respective COG classification was determined using the eggNOG database “bactNOG” (Huerta-Cepas et al., 2016) and is shown for 142 of the 223 CDSs.

Supplementary Table S2 List of 26 USDA 110 genes affected by a frameshift caused by a single nucleotide insertion or deletion compared to strain 110*spc*4. We also list which of the encoded proteins (based on a search against the USDA 110 protein database) and their respective 110*spc*4 orthologs (based on a search against the 110*spc*4 protein database) were found to be expressed. An example of proteogenomics evidence for the protein sequence difference of an annotated CDS is shown in Supplementary Figure S5.

Supplementary Table S3 Lookup “master” table containing 6 sheets with different lists of proteins (corresponding to the results section) from *B. diazoefficiens* 110*spc*4, their locustag identifiers, genomic coordinates, the respective equivalent protein-coding gene (with locustag and coordinates) in *B. diazoefficiens* USDA 110 as well as functional annotations. The “Overview” sheet provides explanations to the individual protein lists; the “Legend” sheet explains the headers of columns shown in individual sheets.

Supplementary Table S4 List of the 91 *B. diazoefficiens* microoxia-induced genes (log_2_ fold change ≥ 1; i.e., FC ≥ 2) whose corresponding protein product was not induced under microoxic conditions compared to oxic conditions (log_2_ FC ≤ 0.5 or multiple testing corrected *p*-value ≥ 0.9).

Supplementary Table S5 List of antibodies and conditions applied in western blot experiments performed in this study.
