# Supplementary figures and images for: An Integrated Systems Approach Unveils New Aspects of Microoxia-Mediated Regulation in Bradyrhizobium diazoefficiens
Source: Front Microbiol. 2019 May 7;10:924. doi: 10.3389/fmicb.2019.00924 (PMC6515984; doi:10.3389/fmicb.2019.00924)

Supplementary Figure S2

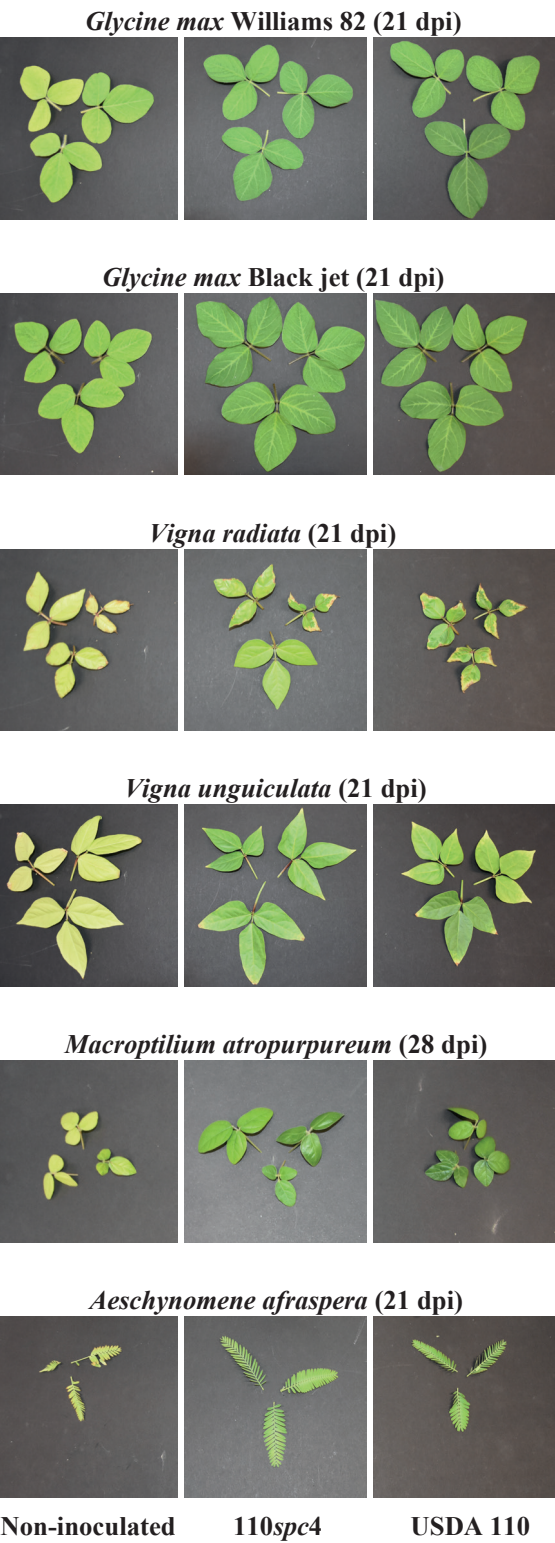

Supplement: Supplementary Figure S2 — Leaf phenotype of different legume hosts inoculated with Bradyrhizobium diazoefficiens 110spc4 or USDA 110. All tested host plants [Glycine max cv. Williams 82 and cv. Black jet (soybeans), Vigna radiata (mung bean), Vigna unguiculata (cowpea), Macroptillium atropurpureum (siratro), and Aeschynomene afraspera] displayed dark-green leaves when inoculated with either 110spc4 (center) or USDA 110 (right) compared to the chlorotic, yellowish leaves of non-inoculated plants (left) typical for nitrogen-starved plants. No qualitative differences between 110spc4- and USDA 110-inoculated plants were visible. Shown are three leaves of three representative plants for each condition. The necrotic spots on V. radiata leaves regularly appear under the given growth conditions, independently of the inoculated strain and do not interfere with symbiosis. [file Data_Sheet_2.PDF]

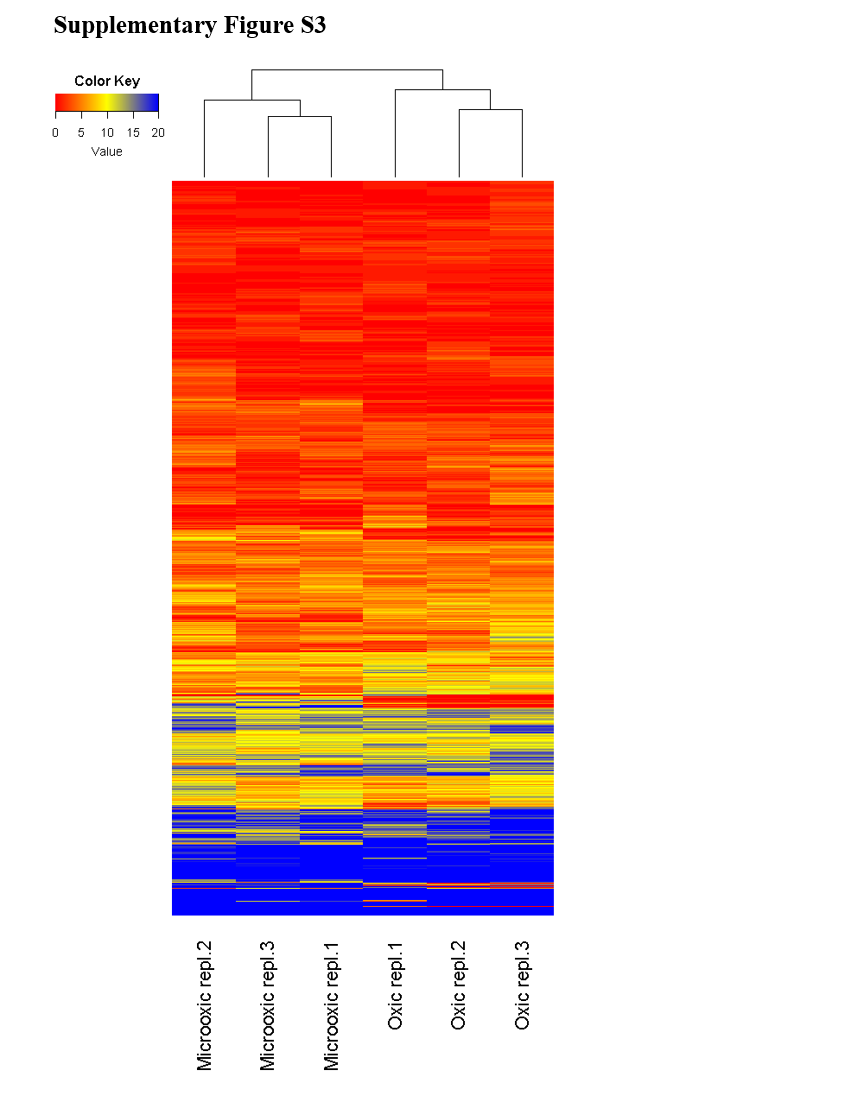

Supplement: Supplementary Figure S3 — Quality assessment of the shotgun proteomics samples (3 biological replicates each for growth under oxic and microoxic conditions) by a clustering analysis (heatmap based on semi-quantitative spectral count data). [file Image_2.PNG]

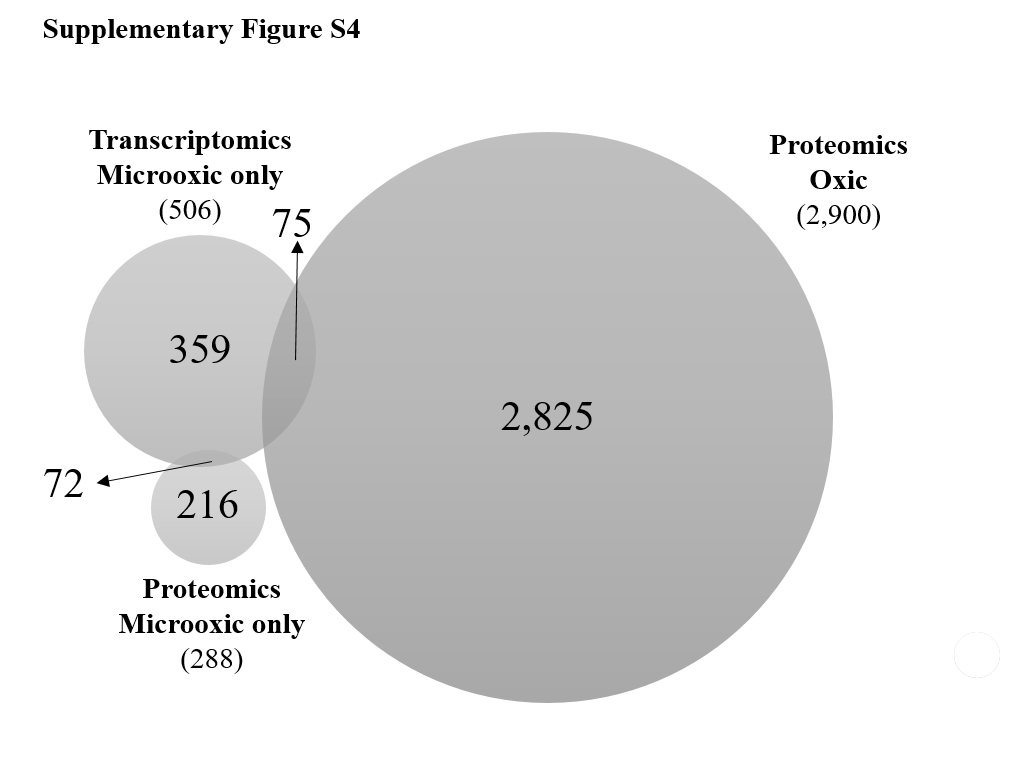

Supplement: Supplementary Figure S4 — Venn diagram showing the overlap of 110spc4 orthologs (see section Materials and Methods) of the 506 previously identified microoxia-specific transcribed genes (Pessi et al., 2007) with the 288 proteins uniquely expressed in microoxia (this study) and 2,900 proteins expressed in oxia (this study). The overlap revealed that 647 genes/proteins were only expressed in microoxia (506-75-72+288). Among these 647, six genes are not present (annotated) in the B. diazoefficiens 110spc4 genome, one is affected by a frameshift (blr6071) and one gene is encoded in the 202 kb deletion region (bsr0039; erroneously implicated to be transcribed likely due to cross-hybridization with cDNA originating from another gene) resulting in 639 microoxia-specific genes/proteins (Supplementary Table S3, Data Sheet E). [file Image_3.PNG]

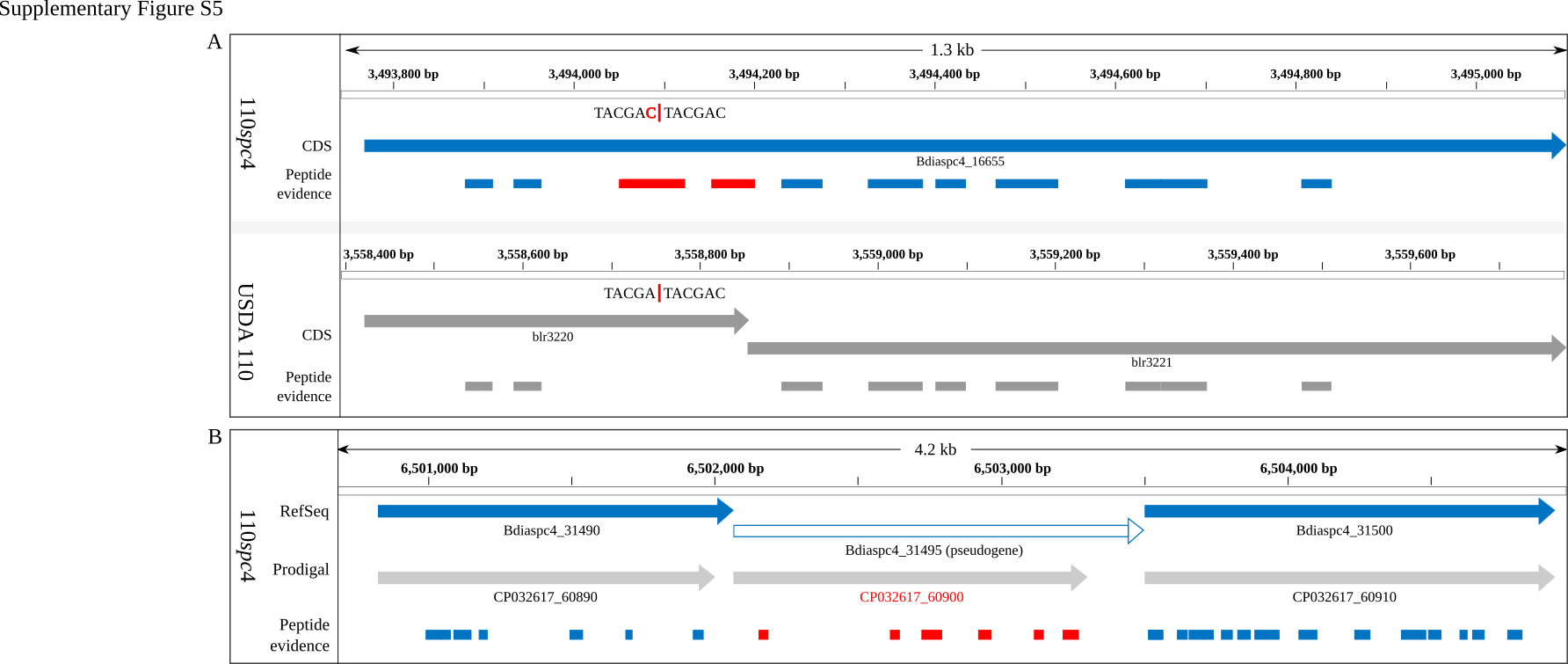

Supplement: Supplementary Figure S5 — Example of proteogenomic evidence for novelties based on genome sequence differences. (A) The correct genomic sequence of strain 110spc4 is shown for a region of interest along with that of the USDA 110 reference strain and the respective annotations. Bdiaspc4_16655 is annotated as a sugar ABC transporter gene in strain 110spc4 (upper panel), predicted to encode a protein of 438 amino acids (blue arrow). A single nucleotide deletion in the genome sequence of strain USDA110 causes a frameshift. Accordingly, two shorter protein-coding genes are annotated (blr3220, blr3221; gray arrows) in the same reading frame. The peptide evidence underlines that our de novo genome assembly is correct: we observed a peptide (red peptide on the left) whose sequence directly confirmed the change compared to USDA 110 and another one traversing the incorrect stop codon (adjacent red peptide). (B) Additional examples can be uncovered using the publicly available iPtgxDB for strain 110spc4. Here, a genomic region of 110spc4 is shown where the NCBI annotation listed two CDSs (Bdiaspc4_31490, Bdiaspc4_31500; blue arrows) and one pseudogene (Bdiaspc4_31495; open arrow). We find proteogenomic evidence for this ORF (red peptides), which was predicted to represent a bona fide CDS by Prodigal (gray boxes; particular gene identifier highlighted in red), underlining the value of such iPtgxDBs to improve the genome annotation of prokaryotic genomes (Omasits et al., 2017). [file Image_4.PNG]
