# Supplementary material for: An Integrated Systems Approach Unveils New Aspects of Microoxia-Mediated Regulation in Bradyrhizobium diazoefficiens
Source: Front Microbiol. 2019 May 7;10:924. doi: 10.3389/fmicb.2019.00924 (PMC6515984; doi:10.3389/fmicb.2019.00924)
Supplement: Supplementary Data Sheet 1 — List of references included in the Supplementary Material. [file Data_Sheet_1.PDF]

Supplementary Data Sheet 1. List of references included in the Supplementary Material.

Bühler, D., Rossmann, R., Landolt, S., Balsiger, S., Fischer, H.M., and Hennecke, H. (2010). Disparate pathways for the biogenesis of cytochrome oxidases in *Bradyrhizobium japonicum*. *J. Biol. Chem.* 285**,** 15704-15713. doi: 10.1074/jbc.M109.085217

Chauhan, S., and O'Brian, M.R. (1995). A mutant *Bradyrhizobium japonicum* delta-aminolevulinic acid dehydratase with an altered metal requirement functions *in situ* for tetrapyrrole synthesis in soybean root nodules. *J. Biol. Chem.* 270**,** 19823-19827. doi: 10.1074/jbc.270.34.19823

Felgate, H., Giannopoulos, G., Sullivan, M.J., Gates, A.J., Clarke, T.A., Baggs, E., et al. (2012). The impact of copper, nitrate and carbon status on the emission of nitrous oxide by two species of bacteria with biochemically distinct denitrification pathways. *Environ. Microbiol.* 14**,** 1788-1800. doi: 10.1111/j.1462-2920.2012.02789.x

Gates, A.J., Hughes, R.O., Sharp, S.R., Millington, P.D., Nilavongse, A., Cole, J.A., et al. (2003). Properties of the periplasmic nitrate reductases from *Paracoccus pantotrophus* and *Escherichia coli* after growth in tungsten-supplemented media. *FEMS Microbiol. Lett.* 220**,** 261-269. doi: 10.1016/S0378-1097(03)00122-8

Grünenfelder, B., Tawfilis, S., Gehrig, S., M, O.S., Eglin, D., and Jenal, U. (2004). Identification of the protease and the turnover signal responsible for cell cycle-dependent degradation of the *Caulobacter* FliF motor protein. *J. Bacteriol.* 186**,** 4960-4971. doi: 10.1128/JB.186.15.4960-4971.2004

Huerta-Cepas, J., Szklarczyk, D., Forslund, K., Cook, H., Heller, D., Walter, M.C., et al. (2016). eggNOG 4.5: a hierarchical orthology framework with improved functional annotations for eukaryotic, prokaryotic and viral sequences. *Nucleic Acids Res.* 44**,** D286-293. doi: 10.1093/nar/gkv1248

Jenal, U., and Fuchs, T. (1998). An essential protease involved in bacterial cell-cycle control. *EMBO J.* 17**,** 5658-5669. doi: 10.1093/emboj/17.19.5658

Jung, S., Yang, K., Lee, D.-E., and Back, K. (2004). Expression of *Bradyrhizobium japonicum* 5-aminolevulinic acid synthase induces severe photodynamic damage in transgenic rice. *Plant Sci.* 167**,** 789-795. doi: 10.1016/j.plantsci.2004.05.038

Kaneko, T., Nakamura, Y., Sato, S., Minamisawa, K., Uchiumi, T., Sasamoto, S., et al. (2002). Complete genomic sequence of nitrogen-fixing symbiotic bacterium *Bradyrhizobium japonicum* USDA110. *DNA Res.* 9**,** 189-197. doi: 10.1093/dnares/9.6.189.

Loferer, H., Bott, M., and Hennecke, H. (1993). *Bradyrhizobium japonicum* TlpA, a novel membrane-anchored thioredoxin-like protein involved in the biogenesis of cytochrome aa_3 and development of symbiosis. *EMBO J.* 12**,** 3373-3383. doi: 10.1002/j.1460-2075.1993.tb06011.x

Mesa, S., Reutimann, L., Fischer, H.M., and Hennecke, H. (2009). Posttranslational control of transcription factor $FixK_2$, a key regulator for the *Bradyrhizobium japonicum*-soybean symbiosis. *Proc. Natl. Acad. Sci. USA* 106**,** 21860-21865. doi: 10.1073/pnas.0908097106

Omasits, U., Varadarajan, A.R., Schmid, M., Goetze, S., Melidis, D., Bourqui, M., et al. (2017). An integrative strategy to identify the entire protein coding potential of prokaryotic genomes by proteogenomics. Genome Res. 27, 2083-2095. doi: 10.1101/gr.218255.116

Pessi, G., Ahrens, C.H., Rehrauer, H., Lindemann, A., Hauser, F., Fischer, H.-M., et al. (2007). Genome-wide transcript analysis of *Bradyrhizobium japonicum* bacteroids in soybean root nodules. *Mol. Plant-Microbe Interact.* 20**,** 1353-1363. doi: 10.1094/MPMI-20-11-1353

Regensburger, B., and Hennecke, H. (1983). RNA polymerase from *Rhizobium japonicum*. *Arch. Microbiol.* 135**,** 103-109. doi: 10.1007/BF00408017

Regensburger, B., and Hennecke, H. (1984). Free-living and symbiotic nitrogen-fixing ability of *Rhizobium japonicum* is unaffected by rifampicin resistance mutations. *FEMS Microbiol. Lett.* 21**,** 77-81. doi: 10.1111/j.1574-6968.1984.tb00189.x

Weiss, M., Schrimpf, S., Hengartner, M.O., Lercher, M.J., and von Mering, C. (2010). Shotgun proteomics data from multiple organisms reveals remarkable quantitative conservation of the eukaryotic core proteome. *Proteomics* 10**,** 1297-1306. doi: 10.1002/pmic.200900414

Yu, N.Y., Wagner, J.R., Laird, M.R., Melli, G., Rey, S., Lo, R., et al. (2010). PSORTb 3.0: improved protein subcellular localization prediction with refined localization subcategories and predictive capabilities for all prokaryotes. *Bioinformatics* 26**,** 1608-1615. doi: 10.1093/bioinformatics/btq249
