# Supplementary material for: An Integrated Systems Approach Unveils New Aspects of Microoxia-Mediated Regulation in Bradyrhizobium diazoefficiens
Source: Front Microbiol. 2019 May 7;10:924. doi: 10.3389/fmicb.2019.00924 (PMC6515984; doi:10.3389/fmicb.2019.00924)
Supplement: Supplementary Table S5 — List of antibodies and conditions applied in western blot experiments performed in this study. [file Table_5.DOCX]

**Supplementary Table S5**. List of antibodies and conditions applied in western blot experiments performed in this study.

| Locus tag  110*spc*4^a^ | Locus tag USDA 110^b^ | Protein^c^ | Protein type | Protein mass (kDa) | Blocking agent | Reducing reagent | Primary antibody against (dilution) | Secondary antibody (dilution) | Source or reference |
| --- | --- | --- | --- | --- | --- | --- | --- | --- | --- |
| *Proteins induced under microoxic conditions compared to oxic conditions* | | | | | | | | | |
| Bdiaspc4_01235 | blr0315 | NosZ | Periplasmic | 71.7 | 1% casein | Dithiothreitol (DTT) | *Paracoccus denitrificans* NosZ (1:1,000) | Anti IgG sheep-HRP (1:3,000) | Felgate et al., 2012 |
| Bdiaspc4_37130 | blr7038 | NapA | Periplasmic | 94.2 | 1% casein | DTT | *P. pantotrophus* M6 NapA (1:1,000) | Anti IgG rabbit-HRP (1:3,000) | Gates et al., 2003 |
| *Proteins not induced under microoxic conditions compared to oxic conditions whose corresponding encoding genes were induced in microoxia in a previous transcriptomics study^d^* | | | | | | | | | |
| Bdiaspc4_05915 | bll1200 | HemA | Soluble | 44.6 | 1% casein | DTT | *Bradyrhizobium diazoefficiens* HemA (1:1,000) | Anti IgG mouse-HRP (1:3,000) | Jung et al., 2004 |
| Bdiaspc4_14260 | bll2757 | FixK_2_ | Soluble | 25.9 | 1% casein | DTT | *B. diazoefficiens* FixK_2_ (1:1,000) | Anti IgG rabbit-HRP (1:3,000) | Mesa et al., 2009 |
| Bdiaspc4_26465 | blr5037 | HemB | Soluble | 63 | 5% nonfat dried milk | Mercaptoethanol | *B. diazoefficiens* HemB (1:2,000) | Anti IgG rabbit-HRP (1:3,000) | Chauhan and O’Brian, 1995 |
| Bdiaspc4_27090 | bll5153 | ClpA | Soluble | 88 | 5% nonfat dried milk | Mercaptoethanol | *Caulobacter crescentus* ClpA (1:1,000) | Anti IgG rabbit-HRP (1:3,000) | Grünenfelder et al., 2004 |
| *Proteins with constant accumulated levels in both oxic and microoxic conditions (controls)* | | | | | | | | | |
| Bdiaspc4_05560 | blr1131 | ScoI | Membrane | 21.3 | 1% casein | DTT | *B. diazoefficiens* ScoI (1:1,000) | Anti IgG rabbit-HRP (1:3,000) | Bühler et al., 2010 |
| Bdiaspc4_05765 | blr1170 | CoxB | Membrane | 30.6 | 5% nonfat dried milk | Mercaptoethanol | *B. diazoefficiens* CoxB (1:10,000) | Anti IgG rabbit-HRP (1:3,000) | Bühler et al., 2010 |
| Bdiaspc4_05770 | blr1171 | CoxA/CtaD | Membrane | 59.3  (migrates as 45) | 1% casein | DTT | *B. diazoefficiens* CoxA (1:1,000) | Anti IgG rabbit-HRP (1:3,000) | Loferer et al., 1993 |
| Bdiaspc4_25960 | bll4944 | ClpP | Soluble | 23.5 | 1% casein | DTT | *C. crescentus* ClpP (1:5,000) | Anti IgG rabbit-HRP (1:3,000) | Jenal and Fuchs 1998 |

^a^ Nomenclature of *B. diazoefficiens* 110*spc*4 genes according to the NCBI annotation (GenBank acc. # CP032617); this work.

^b^ Nomenclature of *B. diazoefficiens* USDA 110 genes according to Kaneko and coworkers (2002) (GenBank acc. # NC_004463; RefSeq annotation from January 2016).

^c^ Protein name according to the UniProt database (https://www.uniprot.org/) with modifications according to functional studies.

^d^ According to Pessi and coworkers (2007).
